# Early initiation of inhaled corticosteroid–long‐acting β_2_‐agonist therapy and reduction of severe asthma exacerbations in high‐risk preschool children: A longitudinal real‐world cohort study

**DOI:** 10.1111/pai.70411

**Published:** 2026-06-14

**Authors:** Arthur H. Owora, Bowen Jiang, Yash Shah

**Affiliations:** ^1^ Division of Pediatric Pulmonology, Allergy/Immunology and Sleep Medicine, Department of Pediatrics Indiana University School of Medicine Indianapolis Indiana USA; ^2^ Center for Biomedical Informatics, Regenstrief Institute Indianapolis Indiana USA

**Keywords:** inhaled corticosteroids, long‐acting β_2_‐agonists, pediatric asthma phenotypes, preschool asthma, risk stratification, severe asthma exacerbations

## Abstract

**Background:**

Evidence supporting inhaled corticosteroid plus long‐acting β_2_‐agonist (ICS + LABA) therapy in preschool children with asthma remains limited, and guideline recommendations remain cautious. However, real‐world effectiveness in high‐risk young children has not been adequately characterized.

**Objective:**

To evaluate short‐ and long‐term effectiveness of ICS + LABA initiation on severe asthma exacerbations (SAEs) and determine whether early‐life asthma risk burden modifies treatment response.

**Methods:**

We conducted a retrospective longitudinal cohort study using electronic health record data from the Indiana Network for Patient Care (2010–2024). Children aged ≤11 years with asthma who initiated ICS + LABA between 2010 and 2023 and had ≥12 months of pre‐ and post‐initiation follow‐up were included. The primary outcome was ≥1 SAE per year, defined per ATS/ERS criteria as asthma‐related hospitalization or emergency department admission. Piecewise generalized linear mixed‐effects models estimated annualized changes in SAE odds before versus after ICS + LABA initiation. Recurrent events were analyzed using Andersen–Gill models. Effect modification by age at diagnosis, age at prior ICS initiation, and early‐life asthma risk burden (passive digital marker [PDM] score) was assessed.

**Results:**

Among 249 children (mean age at asthma diagnosis 2 years; at ICS + LABA initiation 5 years), annualized SAE incidence increased before initiation and declined thereafter. Adjusted SAE odds decreased by 15% per year following ICS + LABA initiation (aOR 0.85; 95% CI 0.58–0.98). Recurrent‐event modeling demonstrated a 68% reduction in SAE hazard after ICS + LABA initiation (aHR 0.32; 95% CI 0.26–0.38), exceeding reductions observed with ICS alone (aHR 0.49). Treatment benefit was greatest among children initiating ICS before age 5 years (aOR 0.77; 95% CI 0.65–0.92) and those with high early‐life asthma risk burden (aOR 0.80; 95% CI 0.67‐0.97). No significant heterogeneity was observed by sex, race, or formulation.

**Conclusions:**

ICS + LABA initiation was associated with sustained reductions in severe exacerbations, including among preschool children, in real‐world clinical practice. These findings suggest that strict age‐based treatment restrictions may not fully capture heterogeneity in exacerbation risk and support further prospective evaluation of risk‐stratified escalation strategies.

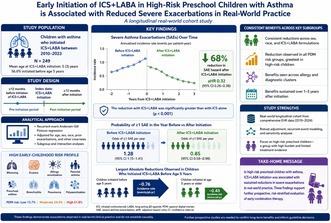

## Graphical abstract

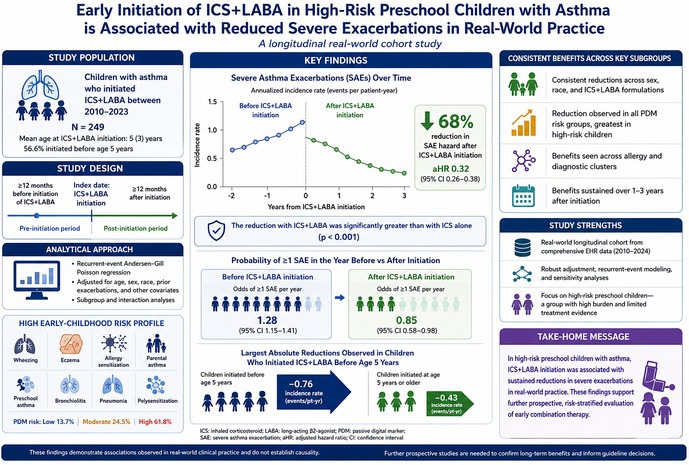

**Abbreviations**
AG: Andersen–Gill
ATS: American Thoracic Society
ED: Emergency Department
EHR: Electronic Health Record
ERS: European Respiratory Society
GINA: global initiative for asthma
GLMM: Generalized Linear Mixed‐Effects Model
HR: hazard ratio
ICS: inhaled corticosteroid
ICS + LABA: Inhaled Corticosteroid plus Long‐Acting β_2_‐Agonist
INPC: Indiana Network for Patient Care
LTRA: leukotriene receptor antagonist
MDC: major diagnostic category
NAEPP: National Asthma Education and Prevention Program
OR: odds ratio
PDM: passive digital marker
RCT: randomized controlled trial
SAE: severe asthma exacerbation

**Key message**

Strict age‐based treatment restriction may overlook high‐risk preschool children in whom early ICS + LABA initiation is associated with reduced severe exacerbations in real‐world practice.

## 1 INTRODUCTION

Evidence on the effectiveness of the use of inhaled corticosteroids (ICS) in combination with long‐acting beta‐agonists (LABA) to reduce the risk of severe childhood asthma exacerbations (SAEs) is growing, but findings remain mixed.^1^, ^2^, ^3^, ^4^ Moreover, there is a paucity of RCTs that evaluate the effectiveness of ICS + LABA in children five‐years and younger (pre‐school age). Only one of three RCTs showed benefit for initiation of fluticasone‐salmeterol (vs fluticasone alone) on SAE risk reduction among children 5‐years and younger.^5^, ^6^, ^7^ Similar mixed results have been reported for observational studies with two out of three retrospective cohort studies showing evidence of more than a 80% reduction in SAEs 3‐month post fluticasone propionate (FP) and salmeterol (SA) treatment.^8^, ^9^, ^10^ The study with a longer (12‐month) post‐treatment follow‐up did not show evidence of reduced SAE risk.^10^

Given the inconclusive evidence of ICS + LABA superiority in this age group, existing GINA guidelines do not recommend ICS + LABA for children 5‐years and younger with moderate‐to‐severe asthma.^11^, ^12^, ^13^, ^14^, ^15^, ^16^, ^17^ Instead, higher doses of ICS and/or add‐on leukotriene receptor antagonists (LTRA) are recommended. However, mixed results have also been observed for children receiving current GINA guidelines recommended treatment. Some studies have shown that for some children treated under existing recommendations (i.e., higher doses of ICS and/or LTRA), the use of bronchodilators and higher serum eosinophils have been associated with better asthma control.^17^ Higher doses of ICS were more effective among Black patients while White non‐Hispanics patients had improved response to LTRA.^18^ Important knowledge gaps persist regarding comparative efficacy of different ICS + LABA drugs, long‐term effectiveness, whether early ICS + LABA treatment initiation can modify the natural course of asthma, and whether the burden of early childhood asthma risk factors impact treatment response.

Evidently, the treatment of children with severe asthma is nuanced as shown by evidence from existing RCTs characterized by non‐linear, short, and long‐term variability response to ICS + LABA therapy and so multiple interconnected factors must be considered in making treatment choices.^1^, ^2^, ^3^, ^4^ Particularly, for children under 5 years old with severe asthma, adding LABA to ICS is an evolving area, showing potential but lacking strong guideline endorsement due to mixed evidence and the challenges associated with diagnostic uncertainty. Moreover, in busy pediatric clinics, active electronic health record (EHR) review to identify prior risk/prognostic factors to inform such treatment decisions can be costly, time consuming, error‐prone, and infeasible.^19^, ^20^, ^21^, ^22^

Therefore, the objective of this study was to (1) characterize short‐ and long‐term response to first‐time exposure to ICS + LABA in young children with asthma, and (2) quantify the clinical benefit of ICS + LABA initiation for the reduction of severe asthma exacerbations. We hypothesized that a younger age of treatment initiation and a higher burden of early childhood asthma risk factors would be associated with more pronounced ICS + LABA treatment benefit.

## 2 METHODS

### 2.1 Study design and data sources

We conducted a retrospective, quasi‐experimental, longitudinal cohort study using electronic health record (EHR) data from the Indiana Network for Patient Care (INPC), a statewide health information exchange comprising data from more than two‐thirds of healthcare institutions in Indiana. The INPC captures inpatient, outpatient, and emergency department (ED) encounters, including diagnoses, medication orders, and prescription records. Use of the INPC reduced the likelihood of incomplete outcome capture due to care received outside a patient's primary healthcare network.

### 2.2 Study cohort

We identified children aged 11 years or younger with asthma who were enrolled in an INPC‐participating institution and had at least one healthcare encounter per year of follow‐up between January 1, 2010, and December 31, 2024 (Figure S1). Children with an incident ICS + LABA prescription between January 1, 2010, and December 31, 2023, were eligible, allowing at least 12 months of post‐initiation follow‐up.

The date of first ICS + LABA prescription defined the index date. The pre‐exposure period was defined as 1–5 years before the index date, and the post‐exposure period as 1–3 years after the index date. Only children with at least 12 months of complete observation both before and after initiation were included in annualized analyses.

### 2.3 Severe asthma exacerbations

The primary outcome was at least one severe asthma exacerbation (SAE), defined according to ATS/ERS criteria as an exacerbation resulting in hospitalization or ED admission.^23^ Oral corticosteroid (OCS) prescriptions not associated with hospitalization or ED encounters were excluded to minimize misclassification, as indication could not be reliably ascertained from structured EHR data. SAEs separated by at least 7 days were treated as distinct events.

To characterize time to ICS + LABA initiation, we evaluated two reference timepoints: first documented asthma diagnosis and first controller prescription. Because inclusion required at least one ICS + LABA prescription, all patients experienced the exposure event of interest.

### 2.4 Covariates

Covariates were identified using ICD‐9/ICD‐10 codes and structured EHR fields (Table S1). Demographic variables included age at asthma diagnosis, sex, and reported race. Clinical history included parental asthma and prior asthma‐related medications (ICS, ICS + LABA, OCS).

Early‐childhood asthma risk burden was quantified using a validated passive digital marker (PDM) score, constructed from EHR data recorded at age 3 years or younger– a period characterized by diagnostic uncertainty.^24^ The PDM incorporates race, parental asthma, eczema, wheeze (with or without colds), pneumonia, bronchiolitis, and allergic sensitization or reported allergies.

To account for heterogeneity in allergic phenotypes, we applied hierarchical clustering on principal components (HCPC)^25^ to reported allergies and specific IgE sensitization results, generating homogeneous allergy clusters. These clusters were evaluated to determine whether allergen sensitization phenotypes modified response to ICS + LABA initiation.^26^ We similarly applied HCPC to pre‐initiation multimorbidity and asthma treatment profiles (e.g., ICS, short‐acting β_2_‐agonists, leukotriene receptor antagonists, antibiotics) to identify health and treatment clusters associated with differential response.

### 2.5 Statistical analysis

We summarized demographic and clinical characteristics before and after ICS + LABA initiation using descriptive statistics. Kaplan–Meier methods were used to characterize time from first asthma diagnosis and first controller prescription to ICS + LABA initiation.

To estimate changes in SAE rates before and after ICS + LABA initiation, we fitted piecewise generalized linear mixed‐effects models (GLMMs), specifying the index year as year 0. These models accounted for repeated measures within individuals and estimated pre‐ and post‐initiation slopes in annualized SAE odds. We tested differences between slopes using linear combinations of model coefficients.

Pre‐specified subgroup analyses were conducted by sex, race, PDM risk category, allergy cluster, treatment cluster, and baseline disease severity. Exploratory models included two‐way and three‐way interaction terms to assess effect modification. In the absence of statistically significant interactions, models were adjusted for potential confounders identified using a directed acyclic graph (DAG) framework.^27^

Sensitivity analyses quantified robustness to unmeasured confounding using *E*‐values.^28^ An E‐value of 1 indicates no unmeasured confounding is required to explain the observed association, whereas an *E*‐value of 2 indicates that an unmeasured confounder would need to double the risk of both exposure (ICS + LABA initiation) and outcome (SAE) to fully account for the association.

To examine recurrent SAE events and time‐varying medication exposure, we fitted Andersen–Gill models,^29^ an extension of the Cox proportional hazards model. This approach treats each SAE as a distinct event within an individual's total at‐risk time (from asthma diagnosis to last observed encounter or end of study). The Andersen–Gill framework accommodates recurrent events, time‐dependent covariates, and complex censoring patterns.

Final models were selected based on the optimal balance between Model fit and parsimony using Akaike and Bayesian information criteria. Two‐sided *p* values <.05 were considered statistically significant. Analyses were conducted in R version 4.4.3.

## 3 RESULTS

### 3.1 Participant characteristics

The cohort comprised 249 children with asthma who initiated ICS + LABA between 2010 and 2023 (Figure S1, Table 1). Of these, 40.6% were female, 39.0% were Black, and 6.4% were Hispanic/Latino; 68.7% were insured by Medicaid. Mean (SD) age at asthma diagnosis and ICS + LABA initiation was 2 (2) and 5 (3) years, respectively. The median follow‐up was 9 years (IQR 5–12).

**TABLE 1:** Demographic and clinical characteristics of the study cohort.

Characteristic	Categories/Statistic	*N* = 249
Sex	Female	101 (40.6%)
	Male	148 (59.4%)
Race	White	139 (55.8%)
	Black/African American	97 (39.0%)
	Others^a^	13 (5.2%)
Ethnicity	Non‐Hispanic/Latino	233 (93.6%)
	Hispanic/Latino	16 (6.4%)
Asthma diagnosis age (years)	Mean (SD)	2 (2)
ICS + LABA Initiation age (years)	Mean (SD)	5 (3)
ICS + LABA initiation <5 years old	*n* (%)	141 (56.6%)
ICS + LABA drug	Fluticasone salmeterol (F/SAL)	158 (63.5%)
	Budesonide Formoterol (BUD/F)	50 (20.1%)
	Mometasone formoterol (M/F)	33 (13.3%)
	Fluticasone vilanterol (F/V)	5 (2.0%)
	More than one drug	3 (1.2%)
ICS drug	Fluticasone (F)	110 (44.2%)
	Budesonide (BUD)	84 (33.7%)
	Beclomethasone (B)	20 (8.0%)
	Mometasone (M)	6 (2.4%)
	More than one ICS	29 (11.6%)
Early‐childhood asthma risk factors used to generate the PDM score	Allergy sensitization^c^	163 (65.5%)
	Eczema^c^	133 (53.4%)
	Wheezing^c^	200 (80.3%)
	Wheezing without cold^c^	194 (77.9%)
	Parental asthma^c^	121 (48.6%)
	Pre‐school asthma	208 (83.5%)
	Bronchiolitis^c^	104 (41.8%)
	Pneumonia^c^	132 (53.0%)
PDM Score/Risk	Low (0–39)	34 (13.7%)
	Moderate (40–79)	61 (24.5%)
	High (80–115)	154 (61.8%)
SAEs 1‐year prior to ICS + LABA	Yes	90 (36.1%)
LTRA 1‐year prior to ICS + LABA	Yes	107 (43.0%)
Allergies/Polysensitization Clusters^b^	1	171 (68.7%)
	2	37 (14.9%)
	3	41 (16.5%)
MDC diagnosis clusters ≤3 years old	1	95 (66.0%)
	2	17 (11.8%)
	3	23 (16.0%)

Abbreviations: B, beclomethasone; BUD, budesonide; BUD/F, budesonide formoterol; F, fluticasone; F/SAL, fluticasone salmeterol; F/V, fluticasone vilanterol; ICS + LABA, inhaled corticosteroid; LABA, long‐acting beta‐agonist; M, mometasone; M/F, mometasone formoterol; PDM, passive digital marker.

^a^ Others in Race: Asian/Pacific Islander 2 (0.8%); Multiracial 1 (0.4%); None of these apply 2 (0.8%); Patient Unavailable 1 (0.4%); Other/Unknown 7 (2.8%).

^b^ All allergies are from NLP source.

^c^ All early‐childhood asthma predictors used to generate the PDM score were determined at ≤3 years of age; ICS + LABA.

Based on the passive digital marker (PDM) score derived from data at age ≤3 years, 86.3% were classified as moderate‐to‐high risk for persistent school‐age asthma. Children with higher PDM risk were diagnosed with asthma at younger ages (Figure S2), although age at ICS/controller initiation did not differ by PDM risk category. All patients had received at least one course of ICS in the year preceding ICS + LABA initiation.

### 3.2 Correlates of ICS + LABA initiation

Fluticasone‐salmeterol was the most prescribed ICS + LABA (63.5%), followed by budesonide‐formoterol (20.1%) and mometasone‐formoterol (13.3%). Initiation before age 5‐years was more frequent among children prescribed fluticasone‐salmeterol and was associated with higher PDM risk and earlier asthma diagnosis (Table S2).

**FIGURE 1:**
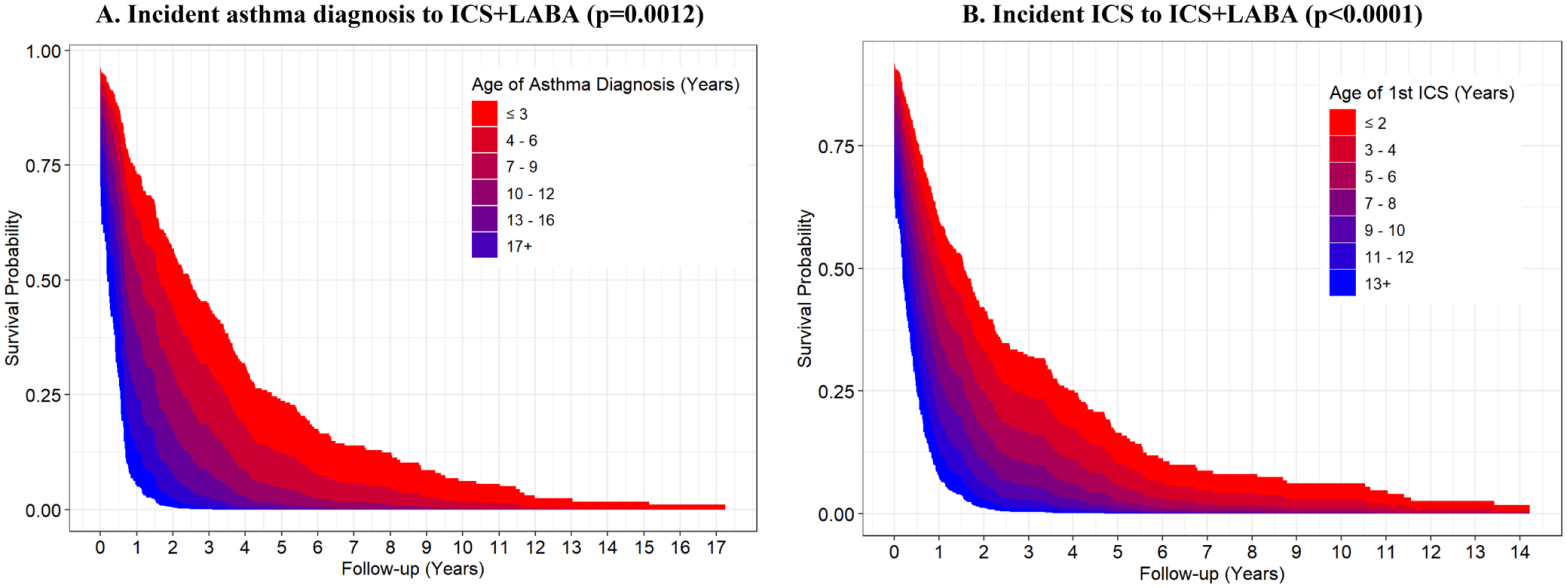
Kaplan–Meir Plot: Time from incident asthma diagnosis (A) OR ICS/controller (B) to ICS + LABA initiation.

Median time from asthma diagnosis to ICS + LABA initiation was 2 years and from first ICS/controller prescription 1‐year (Figure S3). Younger age at asthma diagnosis and ICS initiation was associated with longer time to ICS + LABA initiation (Figure 1; log‐rank test: *p* < .001 for both). In multivariable Cox regression, older age at asthma diagnosis was independently associated with earlier ICS + LABA initiation (adjusted hazard ratio [aHR] 1.44, 95% CI 1.31–1.59), after adjustment for insurance, race, and PDM risk.

Hierarchical clustering identified three allergy clusters explaining 44.3% of cohort variance and three multimorbidity (major diagnostic category [MDC]) clusters explaining 35.0% (Figures S4 and S5, Tables S3 and S4). Allergy clusters distinguished children with low environmental sensitization (Cluster 1) from those with broader aeroallergen (Cluster 2) or food allergy profiles (Cluster 3). MDC clusters differentiated children by burden of early‐life respiratory, neonatal, and social determinant‐related diagnoses (Clusters 1 vs. 2 and 3). These clusters were incorporated into treatment‐response analyses.

### 3.3 Severe asthma exacerbations before and after ICS + LABA initiation

**FIGURE 2:**
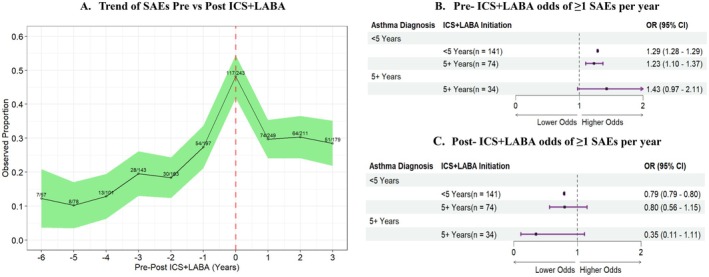
Pre‐Post ICS + LABA initiation Prevalence and Odds of severe exacerbations.

Annualized incidence of severe asthma exacerbations (SAEs) increased progressively in the years preceding ICS + LABA initiation (from <20% to 48%) and declined sharply during the 1–3 years after initiation (Figure 2). In adjusted piecewise mixed‐effects models, the odds of ≥1 SAE decreased by approximately 15% per year following ICS + LABA initiation (adjusted odds ratio [aOR] 0.85, 95% CI 0.58–0.98), controlling for demographic characteristics, PDM risk, and prior asthma treatment duration.

Pre‐post slope differences did not vary significantly by ICS + LABA formulation (interaction *p* > .5). Although reductions were most consistent for fluticasone‐salmeterol and budesonide‐formoterol, differences between drug combinations were not statistically significant (Figure S6).

### 3.4 Heterogeneity of treatment effects

Before ICS + LABA initiation, children diagnosed with asthma before age 5 years had higher annual odds of SAEs than those diagnosed at age 5 years or older (pre‐slope interaction *p* < .001). However, although older children generally demonstrated lower baseline exacerbation risk, the relative post‐initiation treatment‐related reductions did not significantly differ by age group in interaction analyses. Interestingly, the largest absolute reductions in SAEs were observed among children who initiated ICS + LABA before age 5 years (Figure 2).

Treatment effects differed by age at prior ICS initiation (post‐slope interaction *p* = .0312). Only children who had initiated ICS before age 5 years experienced a significant decline in SAEs after ICS + LABA initiation (aOR 0.77, 95% CI 0.65–0.92).

No statistically significant effect modification was observed by sex, race, PDM risk category, allergy cluster, tobacco exposure, or ICS + LABA formulation in interaction models (all *p* > .05).

### 3.5 Subgroup analyses

Across PDM strata, pre‐initiation SAE odds were higher among children with high PDM risk. Post‐initiation reductions were greatest in this group (Table 2, Figure 2), consistent with their greater baseline allergy burden (Figure S4).

**TABLE 2:** Severe asthma exacerbations pre vs. post ICS + LABA initiation.

Characteristic	Subgroup	Slope before ICS + LABA initiation		Slope after ICS + LABA initiation	
		Crude model* OR (95%CI)	Adjusted model^a^ OR (95%CI)	Crude model* OR (95%CI)	Adjusted model^a^ OR (95%CI)
Whole cohort^a^		1.20 (1.20, 1.20)	1.28 (1.15, 1.41)	0.82 (0.81, 0.81)	0.85 (0.58, 0.98) ^#^
Sex^b^	Male	1.19 (1.19, 1.19)	1.32 (1.13, 1.54)	0.85 (0.85, 0.86)	0.79 (0.64, 0.98) ^#^
	Female	1.36 (1.16, 1.60)	1.52 (0.25, 1.85)	0.96 (0.76, 1.20)	0.89 (0.69, 1.15)
Race^c^	White	0.12 (0.98, 1.28)	1.33 (1.11, 1.59)	0.86 (0.69, 1.06)	0.77 (0.60, 0.98) ^#^
	Black	1.34 (1.16, 1.56)	1.45 (1.25, 1.68)	0.97 (0.79, 1.19)	0.91 (0.73, 1.12)
	Other/Unknown	0.93 (0.64, 1.35)	1.19 (0.68, 2.08)	0.81 (0.40, 1.65)	0.66 (0.28, 1.56)
ICS + LABA^d^	Fluticasone salmeterol	1.22 (1.08, 1.38)	1.41 (1.21, 1.64)	0.90 (0.76, 1.07)	0.82 (0.67, 0.99) ^#^
	Budesonide formoterol	1.26 (1.03, 1.54)	1.21 (0.97, 1.52)	0.71 (0.49, 1.03)	0.75 (0.50, 1.14)
	Mometasone formoterol	1.48 (1.05, 2.09)	1.63 (1.06, 2.51)	1.12 (0.73, 1.73)	1.06 (0.65, 1.73)
PDM risk^e^	Low	1.09 (0.81, 1.47)	1.14 (0.82, 1.57)	0.95 (0.59, 1.55)	0.93 (0.55, 1.56)
	Moderate	1.37 (1.15, 1.62)	1.43 (1.17, 1.76)	0.84 (0.62, 1.13)	0.80 (0.58, 1.11)
	High	1.25 (1.11, 1.42)	1.50 (1.28, 1.75)	0.90 (0.76, 1.07)	0.80 (0.67, 0.97) ^#^
Allergy or allergic sensitization at ≤3 years^f^	Yes	1.23 (1.23, 1.24)	1.40 (1.20, 1.63)	0.82 (0.82, 0.82)	0.75 (0.62, 0.91) ^#^
	No	1.33 (1.11, 1.60)	1.45 (1.17, 4.29)	1.06 (0.82, 1.38)	1.01 (076, 1.34)
Allergy Cluster^f^	1	1.27 (1.12, 1.43)	1.39 (1.19, 1.63)	0.66 (0.54, 0.81)	0.62 (0.50, 0.79) ^#^
	2	1.26 (0.96, 1.66)	1.47 (1.07, 2.03)	1.47 (1.04, 2.09)	1.30 (0.88, 1.90)
	3	1.13 (0.92, 1.39)	1.23 (0.96, 1.57)	1.30 (0.97, 1.75)	1.22 (0.87, 1.70)
MDC Diagnosis Cluster^g^	1	1.30 (1.13, 1.49)	1.42 (1.20, 1.68)	1.04 (0.84, 1.29)	0.98 (0.77, 1.25)
	2	1.20 (1.03, 1.40)	1.35 (1.34, 1.35)	0.87 (0.70, 1.07)	0.79 (0.79, 0.80) ^#^
	3	1.24 (1.23, 1.24)	1.49 (0.99, 2.23)	0.58 (0.58, 0.59)	0.52 (0.30, 0.88)
Age of ICS initiation (years)^h^	0–4 years	1.28 (1.28, 1.29)	1.46 (1.28, 1.66)	0.84 (0.84, 0.85)	0.77 (0.65, 0.92) ^#^
	5–11 years	1.38 (1.04, 1.81)	1.44 (1.05, 1.97)	0.96 (0.66, 1.39)	0.94 (0.62, 1.42)
	11+ years	0.85 (0.57, 1.27)	0.92 (0.57, 1.51)	2.31 (1.00, 5.29)	2.45 (0.89, 6.76)

*Note*: The results of the adjusted piecewise GLMM results in each of the listed subgroups. *The crude models include only time‐related variables for estimating pre‐ and post‐slopes, with no covariate adjustments. ^#^Pre vs. post adjusted slopes were significantly different. ^a^The models for all subjects adjusted for sex, race, and age at first ICS + LABA. ^b^Models stratified by sex adjusted for race, and age at first ICS + LABA. ^c^Models stratified by race adjusted for sex and age at first ICS + LABA. ^d,g,h^Models stratified by ICS + LABA type adjusted for sex, race, and age at first ICS + LABA. ^e^Models stratified by PDM risk adjusted for age at first ICS + LABA. ^f^Models stratified by allergy ≤3 years old or allergy cluster adjusted for sex, race, and age at first ICS + LABA. Red font represent a statistically significant increase in the annual odds of SAE before ICS+LABA initiation (*p* 〈 0.05). Blue font represent a statistically significant decrease in the annual odds of SAE after ICS+LABA initiation (*p* 〈 0.05).

In MDC clusters characterized by higher early‐life respiratory and neonatal morbidity (clusters 2 and 3), ICS + LABA initiation was associated with significant annual reductions in SAEs, whereas reductions were not statistically significant in the lower‐burden cluster 1 (Table 2).

Children with early documented allergies (≤3 years) or lower polysensitization burden demonstrated greater relative reductions in SAEs (aOR 0.75, 95% CI 0.62–0.91; and 0.62, 95% CI 0.50–0.79, respectively). Although interaction terms were not statistically significant, reductions were numerically greater among females, White children, and those treated with fluticasone‐salmeterol (Table 2).

### 3.6 Recurrent exacerbations and time‐varying treatment effects

In Andersen‐Gill recurrent‐event models, initiation of ICS alone was associated with a more modest reduction in recurrent SAEs (aHR 0.49, 95% CI 0.42–0.57), whereas ICS + LABA initiation was associated with a larger reduction (aHR 0.32, 95% CI 0.26–0.38) adjusted for demographics, PDM risk, and use of leukotriene receptor antagonists (LTRA). A post hoc comparison of treatment effects between ICS + LABA and ICS alone within the Andersen–Gill recurrent‐event framework showed that the reduction in recurrent SAE hazard associated with ICS + LABA initiation was significantly greater than that observed following ICS alone (*p* < .001). LTRA initiation was not associated with reduced recurrence (aHR 1.04, 95% CI 0.89–1.22).

Higher PDM scores were independently associated with increased SAE recurrence (aHR 1.01 per unit increase Figure S7), and Black children had a 52% higher recurrence rate compared with White children (aHR 1.52, 95%CI 1.32–1.74).

E‐value analyses indicated that an unmeasured confounder would need to be associated with both ICS + LABA initiation and SAE risk by an odds ratio of at least 1.5 to fully explain the observed treatment effects.

## 4 DISCUSSION

In this real‐world longitudinal cohort, initiation of ICS + LABA therapy was associated with sustained and clinically meaningful reductions in severe asthma exacerbations (SAEs), including among children aged 5 years or younger. Benefits were most pronounced in children with early controller initiation and high multidimensional early‐life asthma risk burden.

These findings suggest that strict age‐based treatment restrictions may not fully capture heterogeneity in exacerbation risk among preschool children with severe asthma.^11^, ^12^, ^13^, ^14^, ^15^, ^16^, ^17^ Importantly, absence of large RCT data in preschool populations should not be conflated with absence of benefit. In our heterogeneous real‐world cohort, early combination therapy was associated with substantial reductions in both annualized and recurrent severe exacerbations, including a 68% reduction in recurrent SAE hazard.

Our findings extend prior RCT and observational evidence by demonstrating sustained effectiveness beyond short‐term windows and across diverse phenotypes.^5^, ^6^, ^7^, ^8^, ^9^, ^10^ Earlier retrospective studies reported reductions exceeding 80% within 3 months, whereas longer follow‐up studies showed attenuation.^8^, ^9^, ^10^ In contrast, our data demonstrate durable risk reduction across 1–3 years, suggesting that early combination therapy may provide stable morbidity reduction in high‐risk populations. Importantly, recurrent‐event analyses demonstrated that the reduction in SAE hazard associated with ICS + LABA initiation was significantly greater than that observed following ICS alone, suggesting that the observed reductions were not solely attributable to general controller initiation or background improvements in asthma management.

Crucially, treatment responsiveness was not uniform. Children with high composite early‐life risk burden‐captured using the validated passive digital marker (PDM) score and multimorbidity clustering‐ derived the greatest benefit.^24^, ^25^ This suggests that treatment escalation decisions should be guided by cumulative risk burden rather than age thresholds alone. The lack of consistent effect modification by race, sex, or formulation further supports a risk‐based rather than demographic‐based escalation paradigm. Younger children demonstrated higher baseline exacerbation burden prior to treatment initiation, consistent with known age‐related vulnerability to severe respiratory events in early childhood. However, the largest absolute reductions following ICS + LABA initiation were observed among children treated before age 5 years, suggesting that children at highest baseline risk may also derive the greatest potential benefit from escalation.

While GINA guidance appropriately reflects uncertainty from limited preschool RCTs, our findings align more closely with the conditional recommendations of the National Asthma Education and Prevention Program (NAEPP), which support ICS + LABA use in selected young children with severe disease.^11^, ^12^, ^13^, ^14^, ^15^, ^16^, ^17^, ^30^ Given the morbidity associated with delayed escalation, strict age‐based restriction may inadvertently contribute to preventable emergency healthcare utilization in high‐risk preschoolers.

This study has limitations inherent to observational designs, including potential residual confounding and treatment selection bias. Because preschool wheezing disorders are heterogeneous and many children experience spontaneous improvement with age, our findings should not be interpreted as definitive evidence of causal treatment efficacy. Residual confounding, regression to the mean, and treatment‐selection effects remain possible despite adjustment strategies and recurrent‐event modeling.

Age‐related physiologic maturation and the natural history of early childhood wheezing may partially contribute to declining exacerbation risk over time independent of treatment. However, the progressive increase in SAE incidence observed prior to ICS + LABA initiation, the persistence of reductions after adjustment for age‐related timing variables, and the larger reductions observed relative to ICS alone suggest that maturation effects alone are unlikely to fully explain the observed associations. Moreover, the consistency of our results across modeling strategies—including recurrent‐event Andersen–Gill models—and E‐value sensitivity analyses indicate that substantial unmeasured confounding would be required to nullify observed associations. Drug‐specific comparisons should not be interpreted as head‐to‐head effectiveness estimates.

Taken together, these findings suggest that age alone may be insufficient to fully characterize escalation eligibility in preschool asthma. In high‐risk children with recurrent severe exacerbations despite ICS therapy, earlier ICS + LABA initiation was associated with reduced acute morbidity in real‐world practice. These findings support further prospective evaluation of phenotype‐informed, risk‐stratified escalation strategies to determine whether early combination therapy can alter long‐term asthma trajectories.

## AUTHOR CONTRIBUTIONS

**Yash Shah:** Validation; writing – review and editing. **Arthur H. Owora:** Conceptualization; investigation; funding acquisition; writing – original draft; methodology; validation; visualization; writing – review and editing; software; formal analysis; project administration; data curation; supervision; resources. **Bowen Jiang:** Validation; writing – review and editing.

## FUNDING INFORMATION

This study was in part supported by NIH grants K01HL166436 (AHO) and R03HS029088 (AHO).

## CONFLICT OF INTEREST STATEMENT

All authors declare no competing interests.

## ETHICS STATEMENT

This study was approved by the Indiana University Institutional Review Board (15869). Requirement for informed consent was waived because of the use of de‐identified electronic health record data.

## Supporting information


**Figure S1.** Study design schematic and flow chart.
**Figure S2.** Kaplan–Meir Plots of time birth to asthma diagnosis (A) to ICS (B) by PDM risk.
**Figure S3.** Kaplan–Meir Plots of time to ICS + LABA from Incident Diagnosis (A/C) or ICS (B/D).
**Figure S4.** Allergy clusters derived from 13 allergy categories based on specific IgE.
**Figure S5.** Major diagnostic category clusters derived from EHR data among children (≤3 years old).
**Figure S6.** Cumulative incidence of SAEs Pre‐Post ICS + LABA by drug formulation.
**Figure S7.** Longitudinal heatmap of SAE recurrence during study follow‐up after an incident asthma diagnosis by PDM Risk.
**Table S1.** Definitions and descriptions of study variables.
**Table S2.** Demographic and clinical characteristics of the study cohort by incident ICS + LABA drug.
**Table S3.** Allergy/Allergy sensitization by derived HCPC clusters.
**Table S4.** MDC diagnoses (≤3 years) by HCPC derived clusters.

## Data Availability

De‐identified individual participant data will be available upon reasonable request to the corresponding author, subject to institutional data use agreements and ethical approvals.
