# Supplementary material for: Early initiation of inhaled corticosteroid–long‐acting β2‐agonist therapy and reduction of severe asthma exacerbations in high‐risk preschool children: A longitudinal real‐world cohort study
Source: Pediatr Allergy Immunol. 2026 Jun 14;37(6):e70411. doi: 10.1111/pai.70411 (PMC13265844; doi:10.1111/pai.70411)
Supplement: Supplementary file 1 — Figure S1. Study design schematic and flow chart. Figure S2. Kaplan–Meir Plots of time birth to asthma diagnosis (A) to ICS (B) by PDM risk. Figure S3. Kaplan–Meir Plots of time to ICS + LABA from Incident Diagnosis (A/C) or ICS (B/D). Figure S4. Allergy clusters derived from 13 allergy categories based on specific IgE. Figure S5. Major diagnostic category clusters derived from EHR data among children (≤3 years old). Figure S6. Cumulative incidence of SAEs Pre‐Post ICS + LABA by drug formulation. Figure S7. Longitudinal heatmap of SAE recurrence during study follow‐up after an incident asthma diagnosis by PDM Risk. Table S1. Definitions and descriptions of study variables. Table S2. Demographic and clinical characteristics of the study cohort by incident ICS + LABA drug. Table S3. Allergy/Allergy sensitization by derived HCPC clusters. Table S4. MDC diagnoses (≤3 years) by HCPC derived clusters. [file PAI-37-e70411-s001.docx]

**Figure S1. Study Design Schematic and Flow Chart**

1. **Schematic design**

**
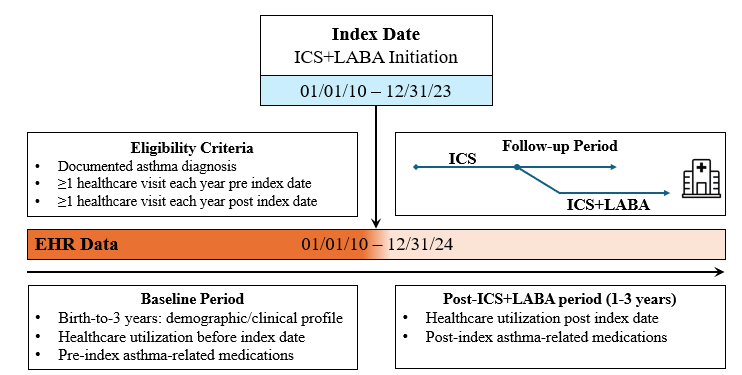
**

1. **Flowchart**

Children with asthma who initiate ICS+LABA between 1/1/2010 and 12/31/2023

**N=273**

**Exclusion criteria**

No prior ICS before index date -24

**Analysis Sample**

**N=249**

Encounter data accrued between January 1st, 2010, through December 31st, 2024, were examined to allow for a reasonable duration of patient follow-up period before and after ICS+LABA initiation. The longer pre (vs post) index period was considered to ensure sufficient data was available to characterize early-childhood asthma risk factors (≤3 years of age), history of disease severity, and prior treatments.

**Figure S2. Kaplan-Meir Plots of time birth to asthma diagnosis (A) to ICS (B) by PDM risk**

| 1. **Birth to Incident Asthma Diagnosis** | 1. **Asthma Diagnosis to Incident ICS** |
| --- | --- |
| 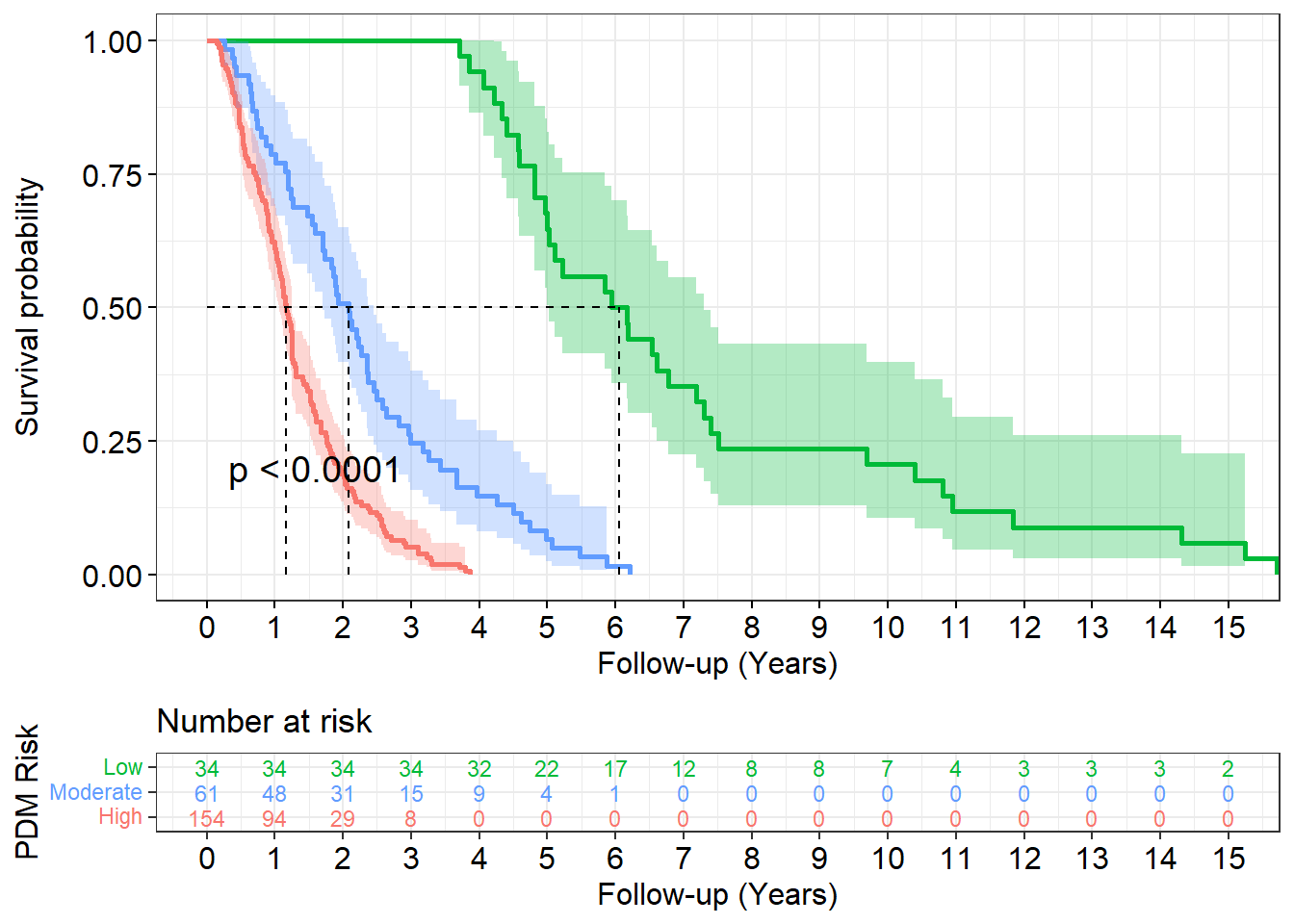 | 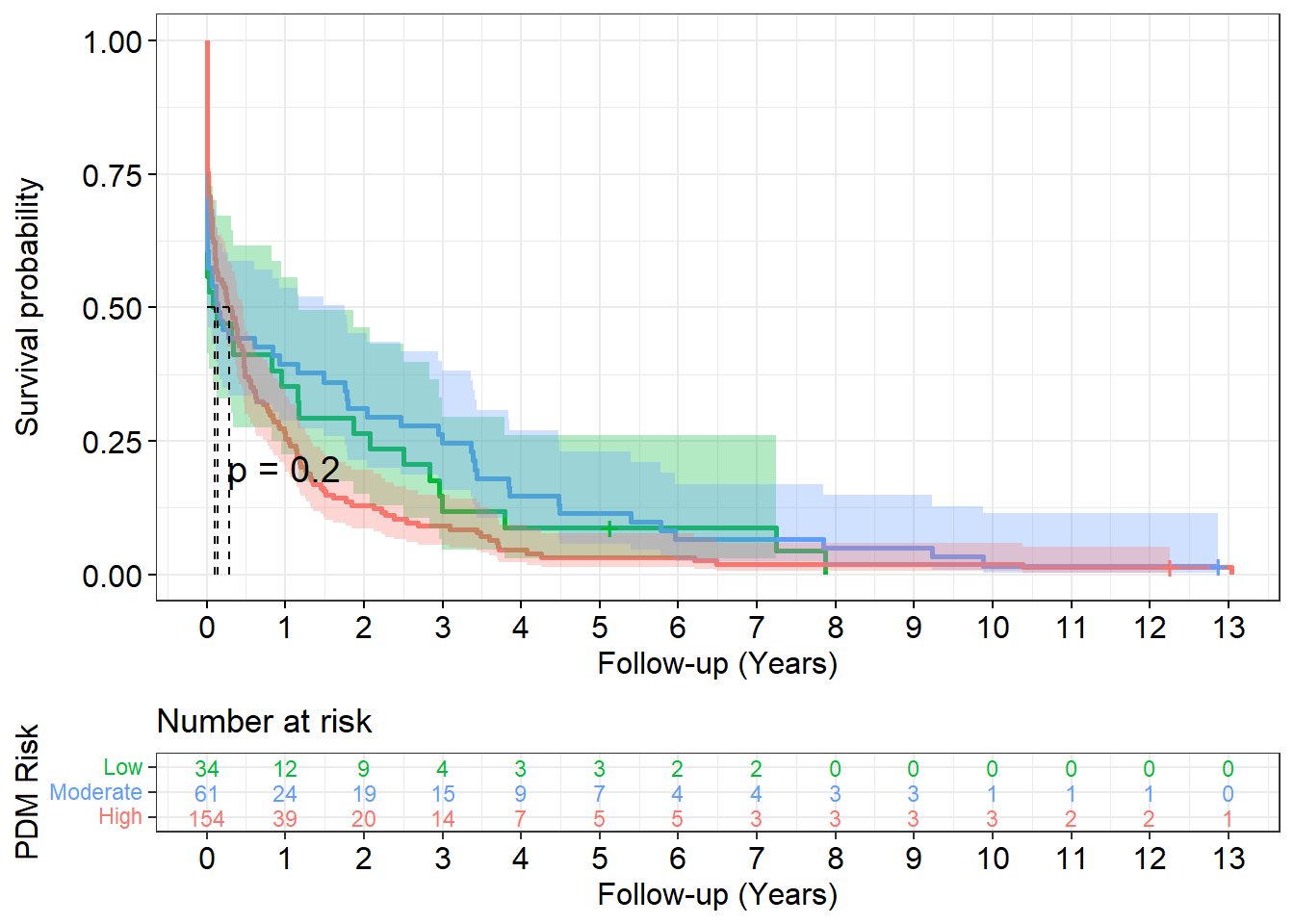 |

**Figure S3. Kaplan-Meir Plots of time to ICS+LABA from Incident Diagnosis (A/C) or ICS (B/D)**

| 1. **Overall: Incident Asthma Diagnosis to ICS+LABA** | 1. **Overall: Incident ICS to ICS+LABA** |
| --- | --- |
| 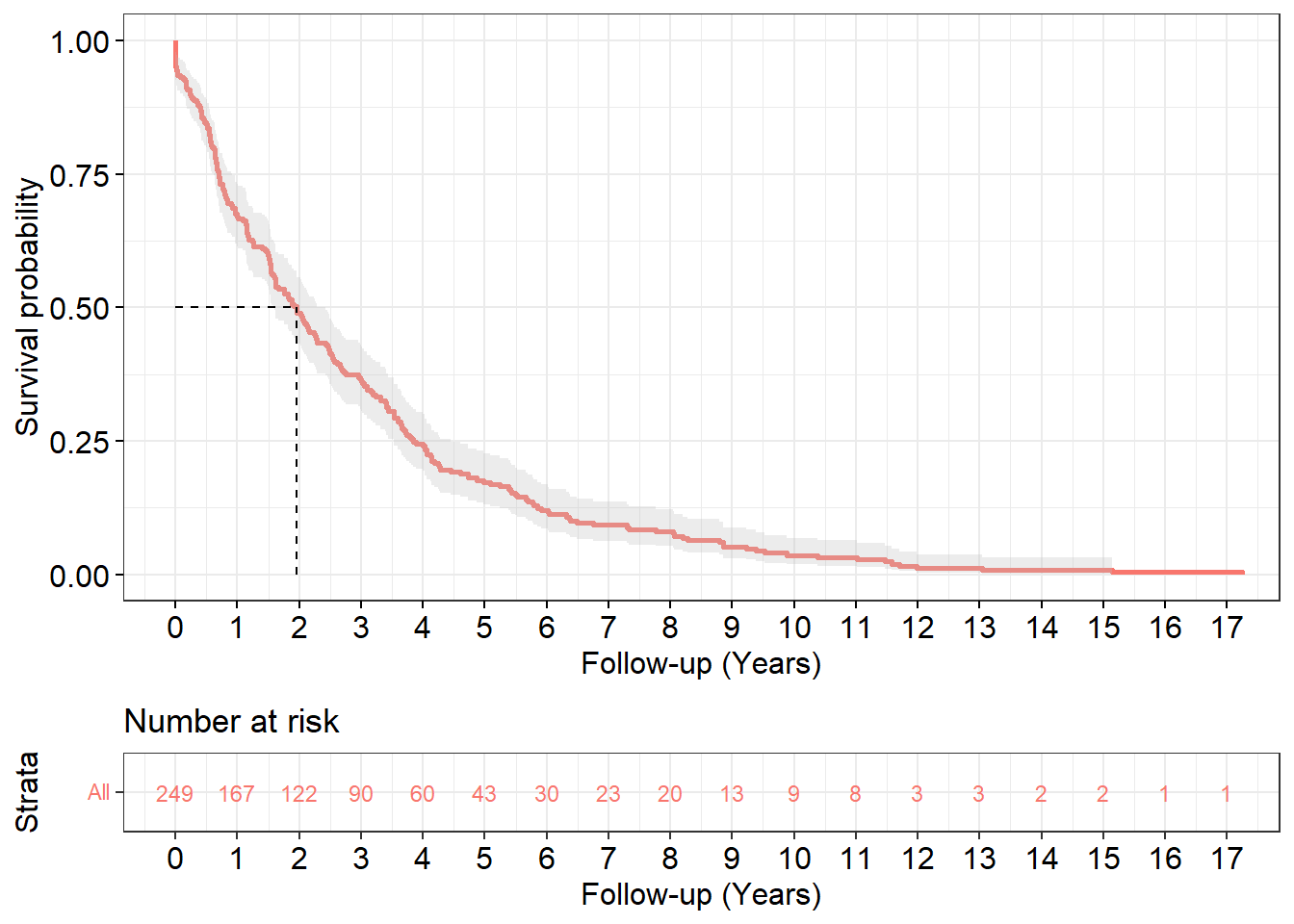 | 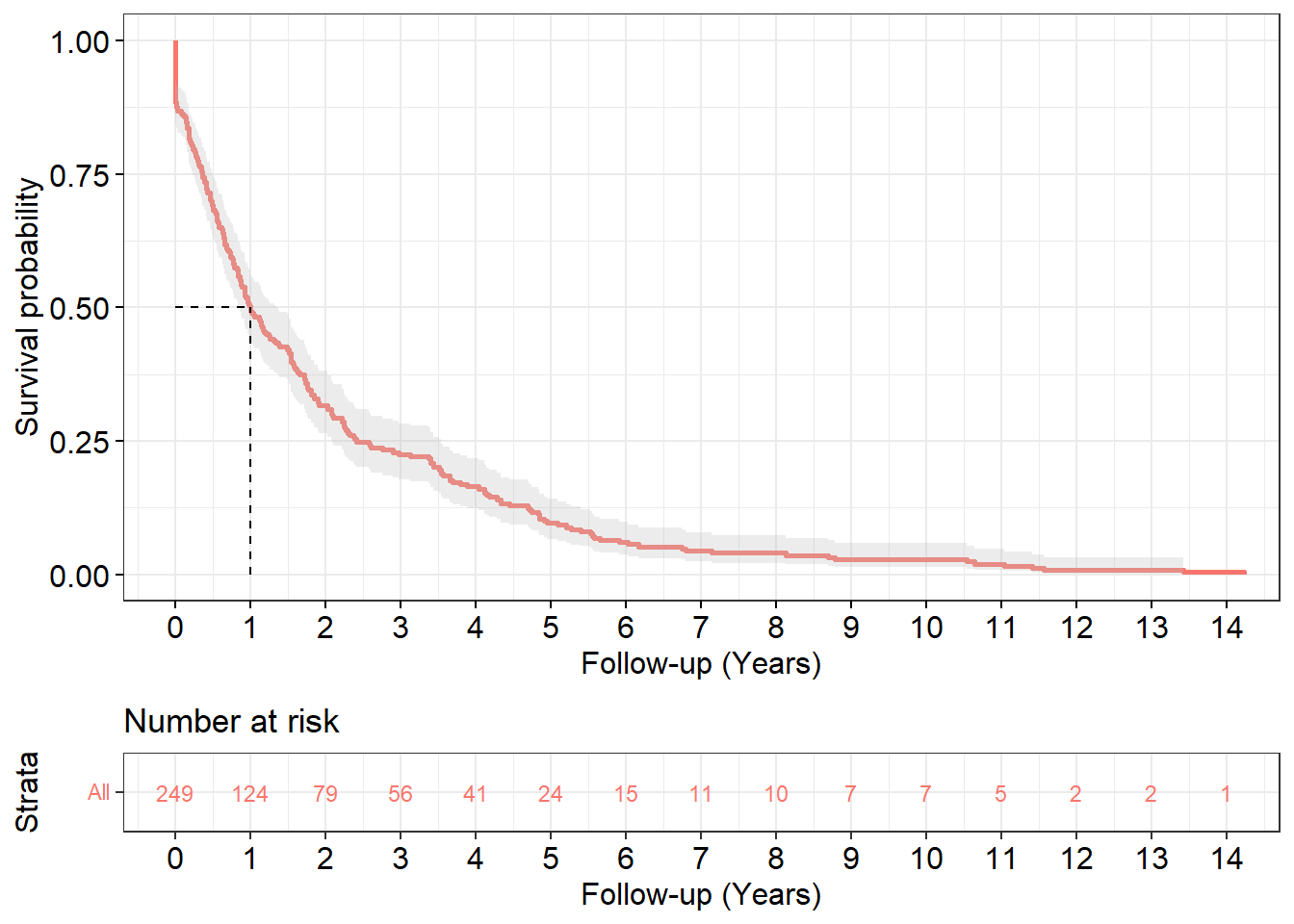 |
| 1. **PDM Risk: Incident Asthma Diagnosis to ICS+LABA** | 1. **PDM Risk: Incident ICS to ICS+LABA** |
| 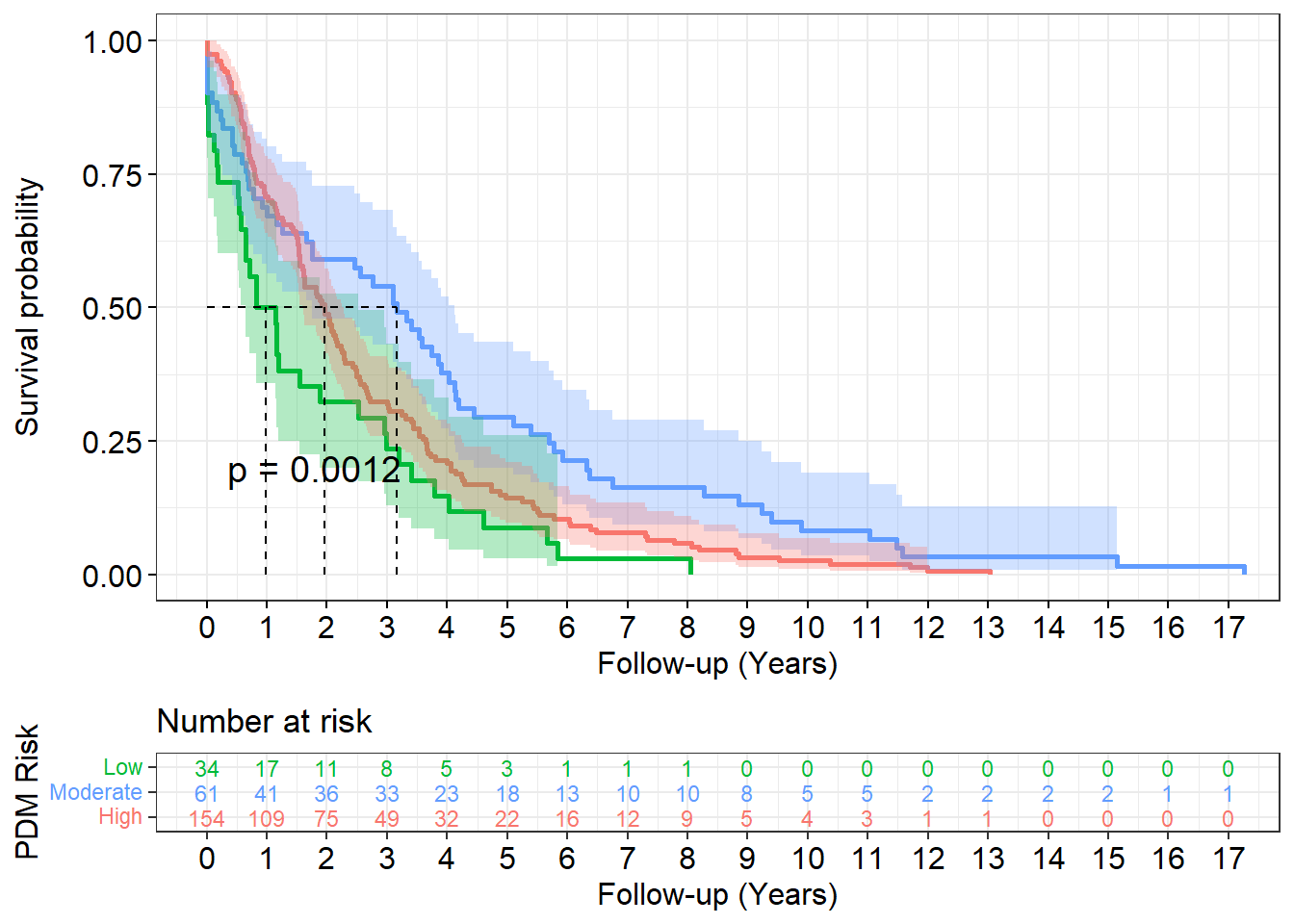 | 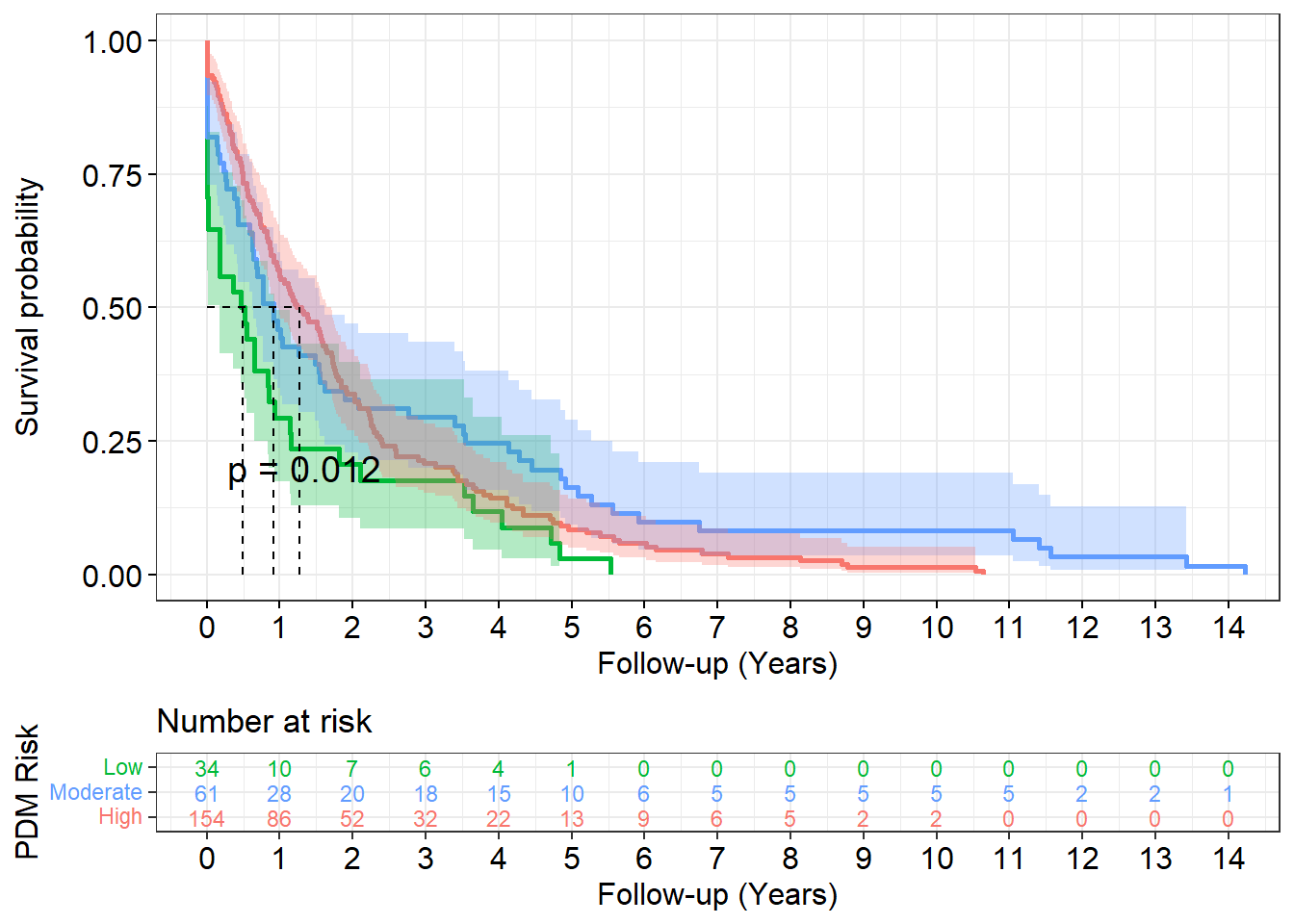 |

**Figure S4. Allergy clusters derived from thirteen allergy categories based on specific IgE**

1. Patients within clusters on the factor map **B.** Sensitization categories by cluster


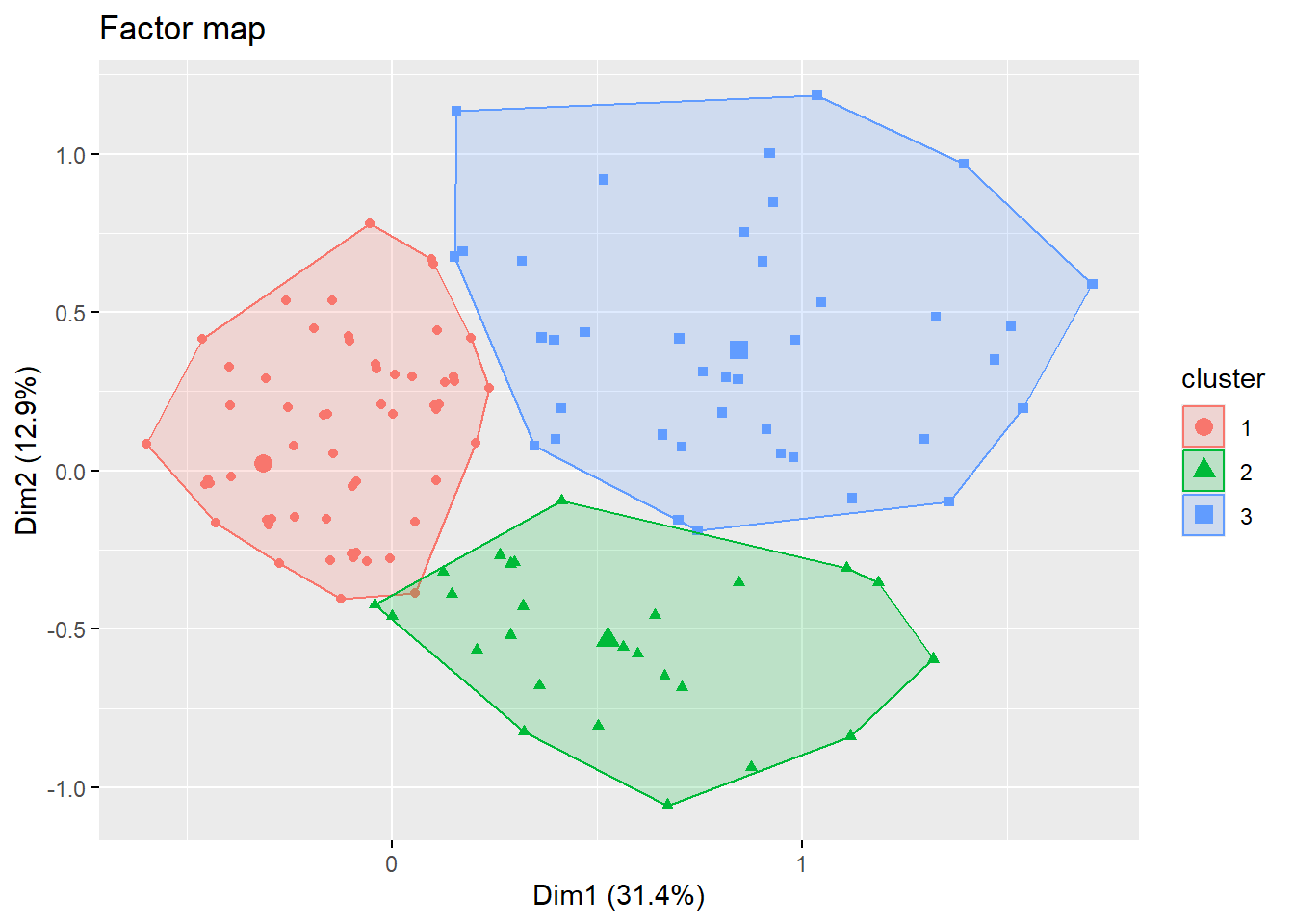

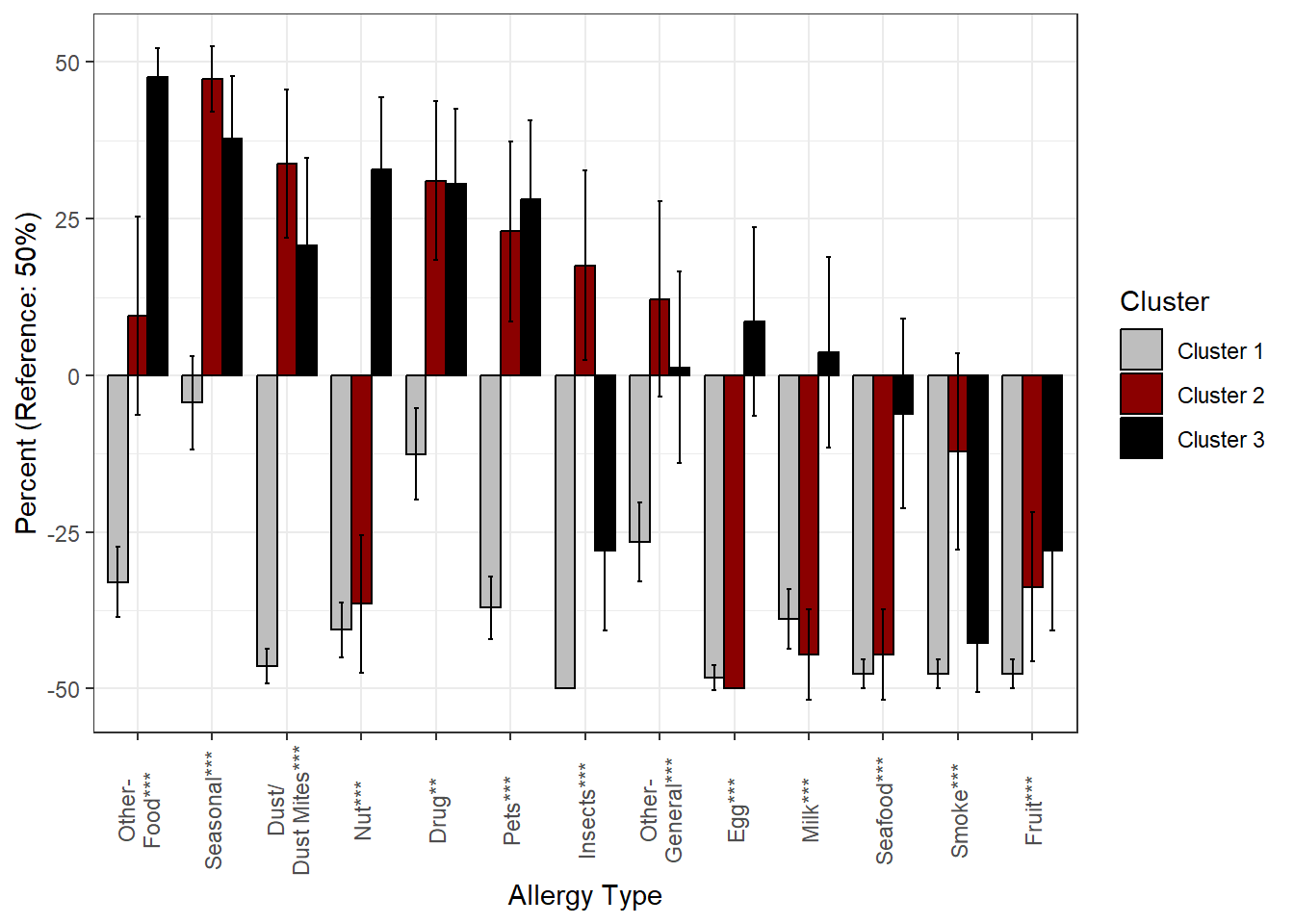


1. Spatial representation of allergy categories **D.** Early-life asthma risk factors by cluster


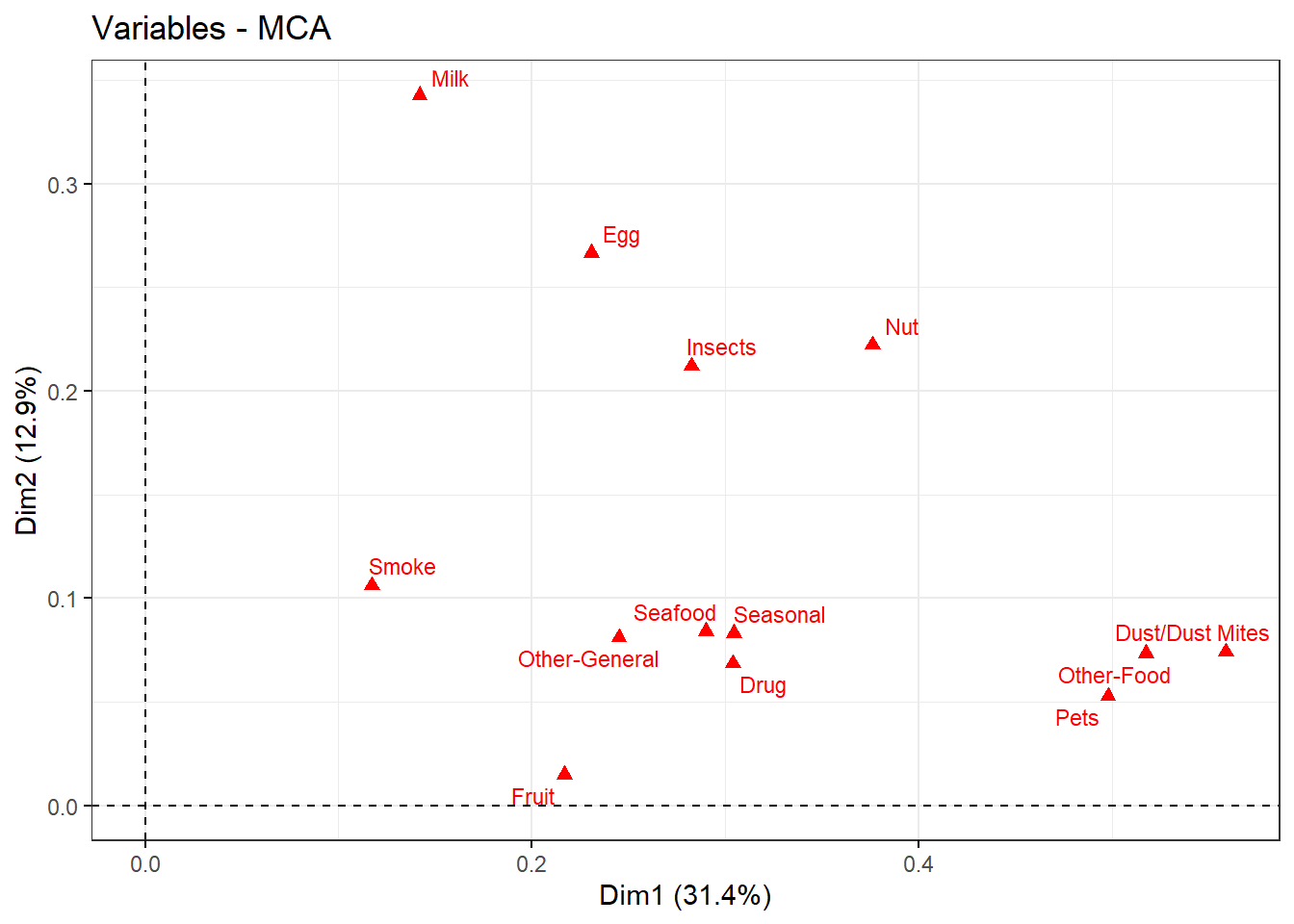

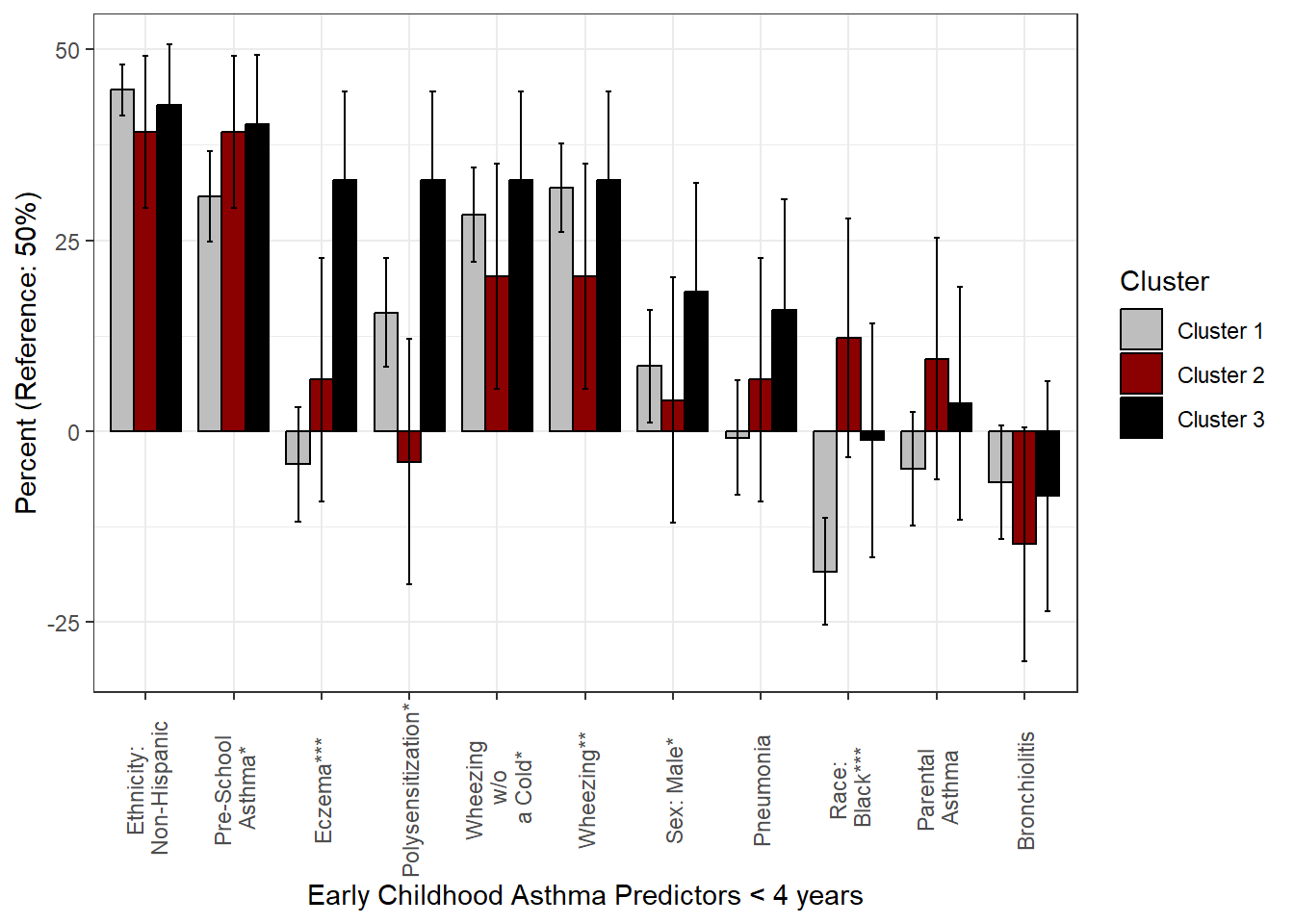


**Figure S5. Major Diagnostic Category clusters derived from EHR data among children (≤ 3 years old)**

1. Patients within clusters on the factor map **B.** MDC categories by cluster


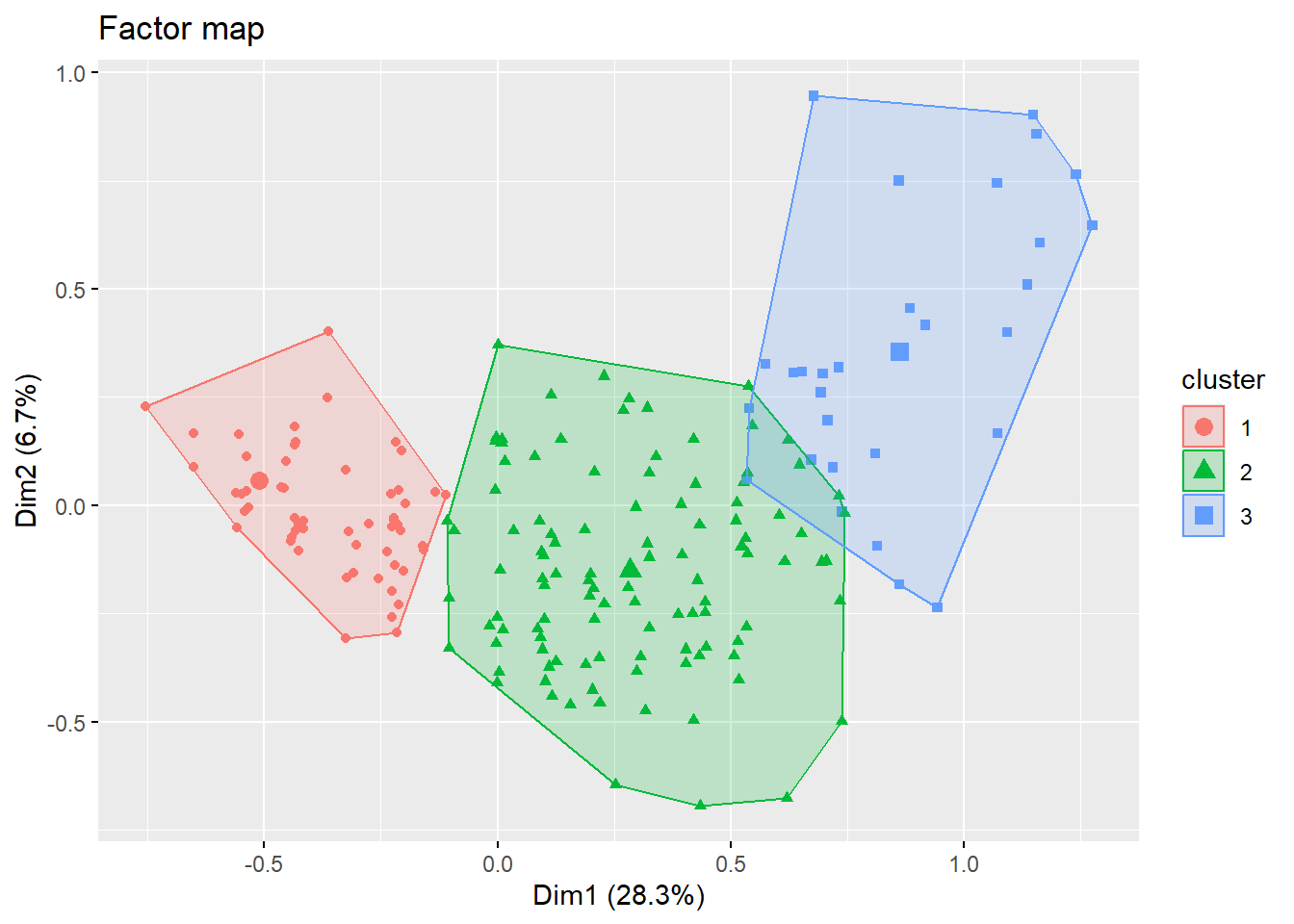

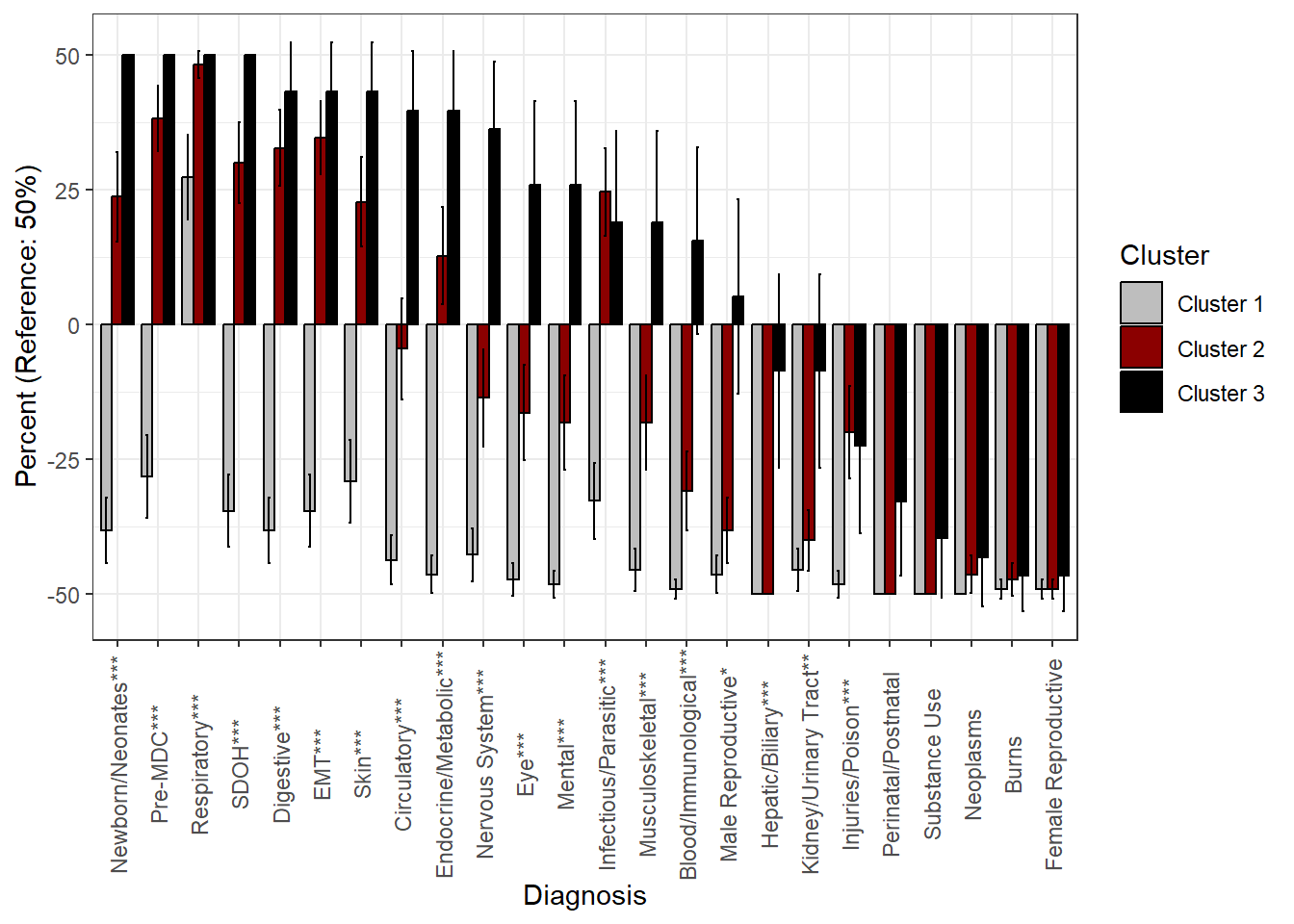


1. Spatial representation of MDC categories **D.** Early-life asthma risk factors by cluster


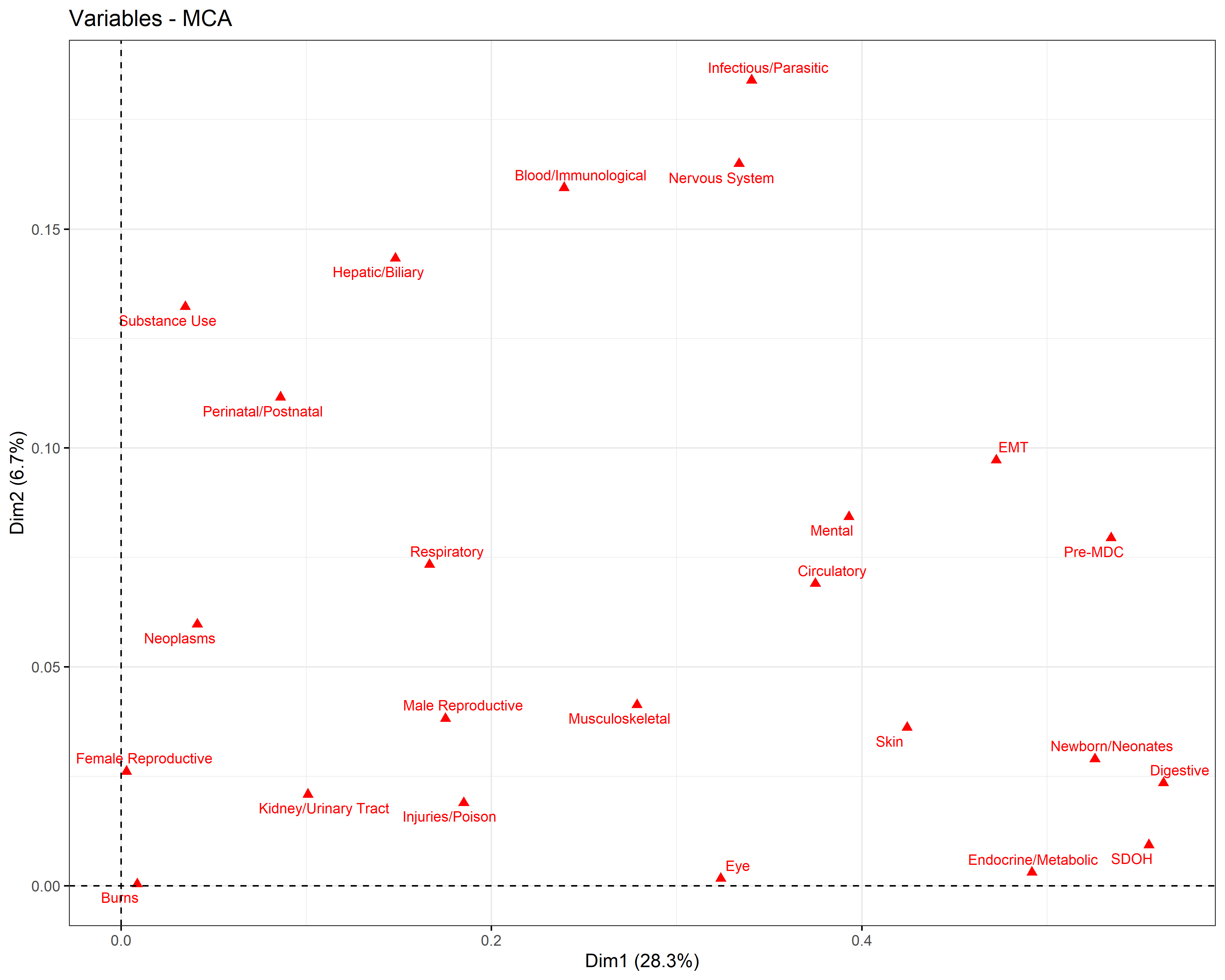

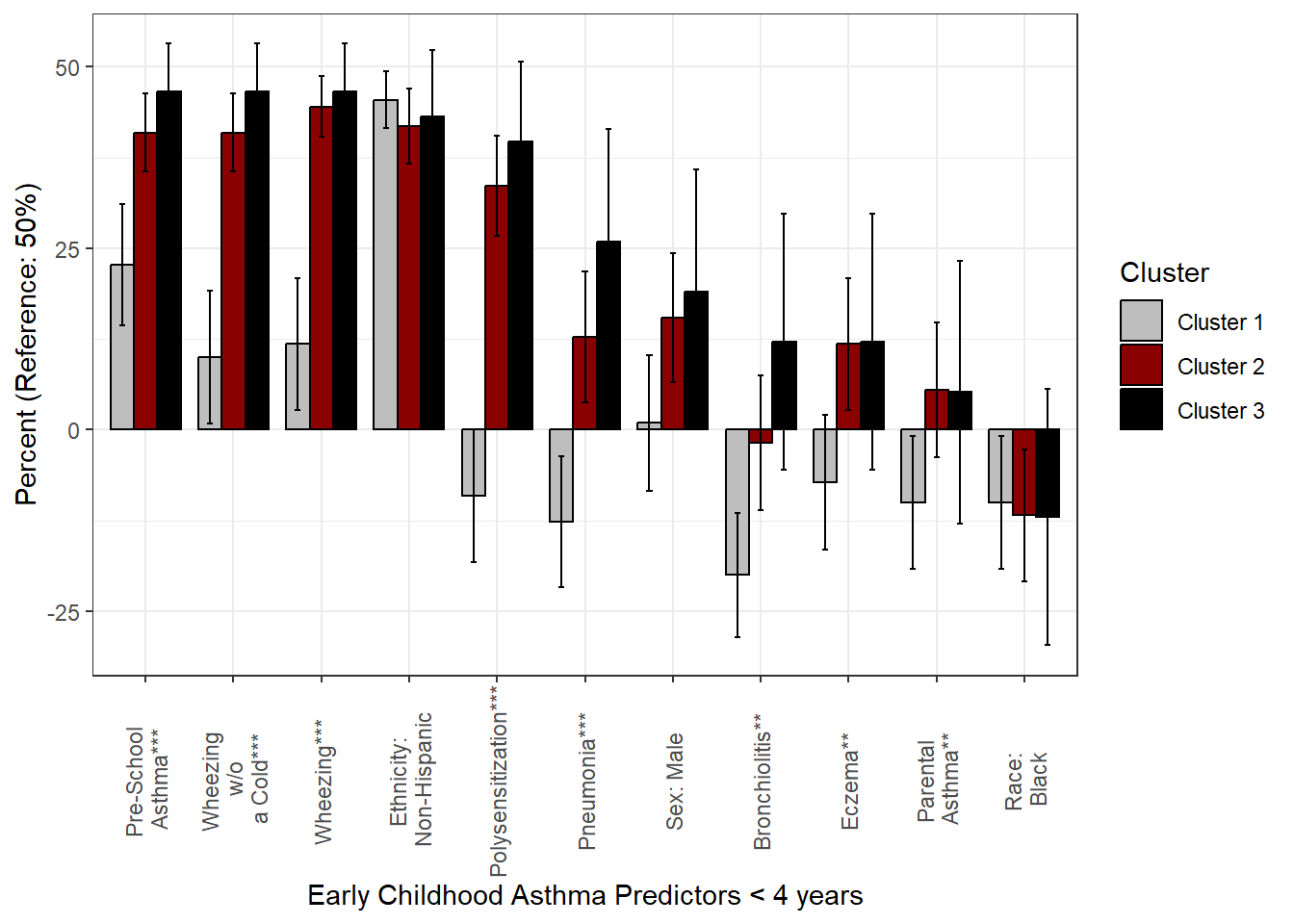


**Figure S6. Cumulative Incidence of SAEs Pre-Post ICS+LABA by Drug Formulation**


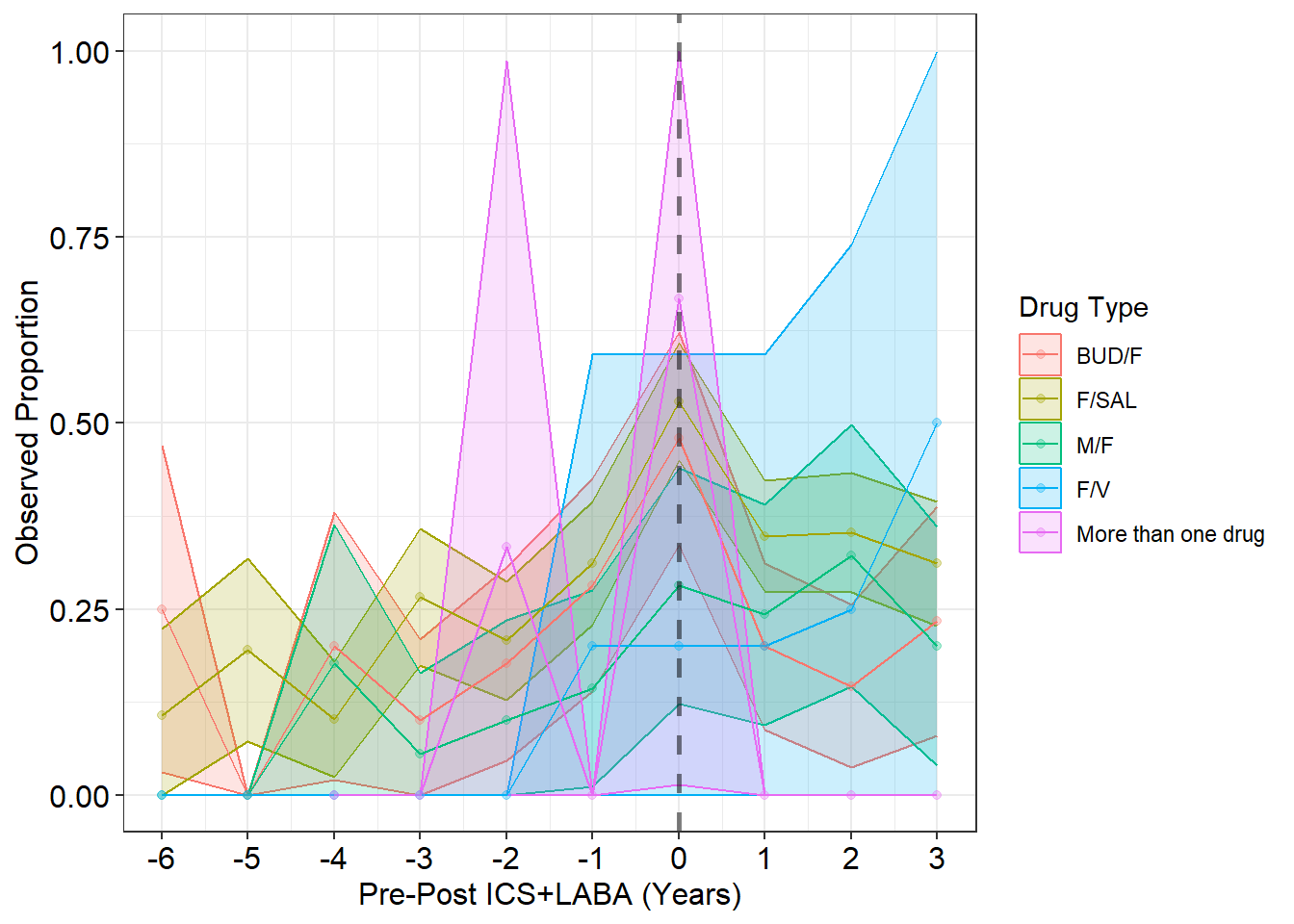


ICS+LABA formulations: budesonide formoterol (BUD/F); fluticasone salmeterol(F/SAL); mometasone formoterol(M/F); fluticasone vilanterol(F/V)

**Figure S7. Longitudinal Heatmap of SAE Recurrence during study follow-up after an incident asthma diagnosis by PDM Risk**


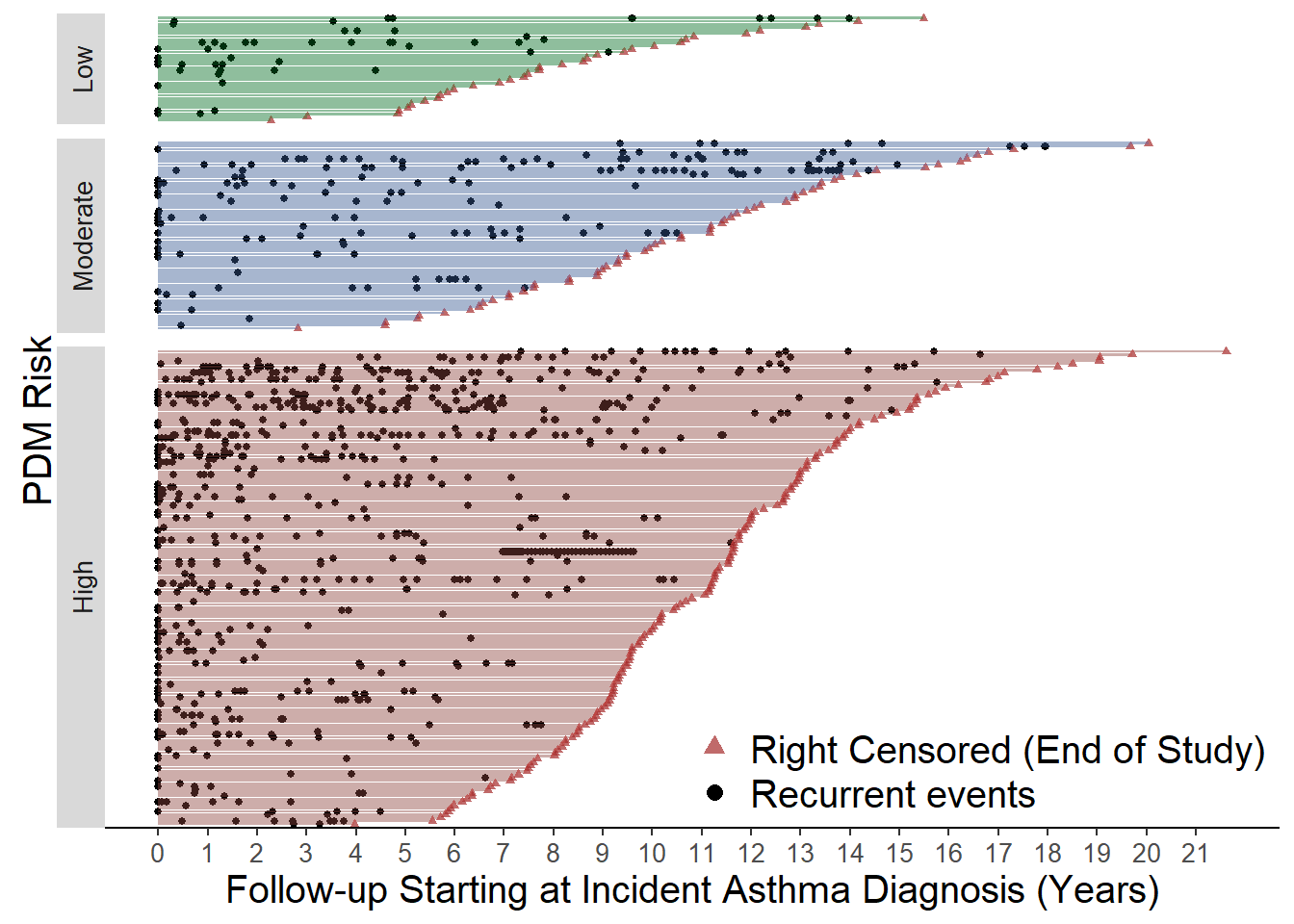


**Table S1. Definitions and descriptions of study variables**

| **Characteristics** | Early childhood predictors of asthma | |
| --- | --- | --- |
| **Key Terms** | Passive digital marker (PDM) | ICD Codes |
| Asthma Diagnosis | Physician documented asthma diagnosis | J45.xx, 493.xx |
| Pre-school age asthma | Physician documented asthma diagnosis ≤ 3 years old | J45.xx, 493.xx |
| Black or African American Race | Documented self-report |  |
| Parental History of Asthma | Documented self-report | Z82.5, V17.5 |
| Wheezing | Physician documented wheeze symptoms/diagnosis ≤ 3 years old | 786.07, J80, R09.2, J96.xx, 518.82, 518.81, 799.1, R06.2 |
| Wheezing without Cold | Physician documented wheeze symptoms /diagnosis that **did not** include cold related symptoms/diagnosis ≤ 3 years old | J00, J31.1, J06.0, J06.9, 465.9, 460 |
| Eczema | Physician documented diagnosis of Eczema ≤ 3 years old | 692.5, 692.6, L30.x, L20 |
| Polysensitization/  Multiple allergies^1^ | Two or more physician documented allergy diagnosis OR Two or more laboratory results of positive allergy sensitization related to aeroallergens or foods based on skin prick test identified as an allergen wheal was ≥3 mm greater than the saline control or a positive allergy blood test of specific immunoglobulin E (IgE) ≥0.35 kU/l. OR At least one allergy diagnosis and one positive allergy sensitization result related to aeroallergens or foods | Z91.0, 995.3, T78.40XA |
| HCPC clusters^2^ | Created from the (1) allergy/allergy sensitization results, (2) MDC diagnosis categories, and (3) asthma-related medications |  |
| Pneumonia | Physician documented of a pneumonia diagnosis ≤ 3 years old | 481, 482, 483, 484, 485, 486, 487.0, J12, J13, J14, J15, J16, J17, J18 |
| Bronchiolitis | Physician documented a bronchiolitis diagnosis ≤ 3 years old | 466.1, 466.11, 466.19, J21.0, J21.1, J21.8, J21.9 |

^1^Descriptors of allergies/polysensitization documented in the EHR: (1) Pollen allergies include allergy to elm, pollen; (2) Dust/Dust Mites allergies include allergy to dust, dust mites, and dander; (3) Nut allergies include allergy to almonds, cashews, pistachios, pecans, peanuts, tree nuts, walnuts, and nuts; (4) Egg allergies include allergy to eggs; (5) Seafood allergies include allergy to crab, lobster, shrimp, fish, shellfish, swordfish, tuna, scallops, and seafood; (6) Seasonal allergies include allergy to fall, winter, autumn, summer, and seasonal; (7) Milk allergies include allergy to milk, and dairy; (8) Fruit allergies include allergy to banana, coconut, pineapple, kiwi, raspberry, strawberry, watermelon, mango and fruits; (9) Pet allergies include allergy to cats, dogs, mice, rodents, pet dander, animal dander, and animals; (10) Drug allergies include allergy to ibuprofen, amoxicillin, Augmentin, Tylenol, erythromycin, antibiotics, penicillin, propylene glycol and drugs; (11) Grass allergies include allergy to grass, and hay; (12) Insects allergies include allergy to wasps, cockroaches, insect stings, bees, hornets and insects; (13) Smoke allergies include allergy to smoke; (14) Mold allergies include allergy to mold; (15) Fur allergies include allergy to fur; (16) Trees/Plants allergies include allergy to birch, oak, pigweed, ragweed, trees and plants; (17) Other-Food allergies include allergy to wheat, gluten, rice, pork, soy, sunflower seeds and foods; (18) Other-General allergies include allergy to adhesive bandages, adhesives, dyes, environment, latex, metals, narcotics, perfumes, and other.

^2^HCPC is a robust method that combines three standard multivariate data analysis methods (Multiple correspondence analysis or principal component analysis for categorical variables, Hierarchical clustering, and K-means clustering) to produce statistical clusters based on patient attributes. An agglomerative algorithm was used to determine the optimal number of clusters that minimized the total intra-cluster variation (i.e., total within-cluster variation or total within-cluster sum of square). To assess convergent validity of the derived clusters, univariate tests (Analysis of variance/Kruskal-Wallis test or Chi-square/Fisher’s exact tests) were used to test for dependence between allergy categories, early childhood asthma risk factors, and derived cluster groups.

**Table S2: Demographic and clinical characteristics of the study cohort by incident ICS+LABA drug**

| **Characteristic** |  | Budesonide Formoterol (BUD/F) N = 50 | Fluticasone Salmeterol (F/SAL) N = 158 | Mometasone Formoterol (M/F) N = 33 | Fluticasone Vilanterol (F/V) N = 5 | **p-value** |
| --- | --- | --- | --- | --- | --- | --- |
| **Sex** | Female | 21 (42.0%) | 59 (37.3%) | 17 (51.5%) | 2 (40.0%) | 0.491 |
| **Race** | White | 30 (60.0%) | 82 (51.9%) | 21 (63.6%) | 3 (60.0%) | 0.244 |
|  | Black | 19 (38.0%) | 68 (43.0%) | 9 (27.3%) | 1 (20.0%) |  |
|  | Other^1^ | 1 (2.0%) | 8 (5.1%) | 3 (9.1%) | 1 (20.0%) |  |
| **Ethnicity** | Hispanic/Latino | 2 (4.0%) | 10 (6.3%) | 3 (9.1%) | 1 (20.0%) | 0.328 |
| **Healthcare Insurance** | Medicaid | 26 (55.3%) | 109 (70.3%) | 27 (81.8%) | 3 (60.0%) | 0.062 |
| **Age at Asthma Diagnosis** | 0-4 Years | 44 (88.0%) | 147 (93.0%) | 26 (78.8%) | 3 (60.0%) | 0.009 |
|  | 5-11 Years | 5 (10.0%) | 10 (6.3%) | 7 (21.2%) | 1 (20.0%) |  |
|  | 11+ Years | 1 (2.0%) | 1 (0.6%) | 0 (0.0%) | 1 (20.0%) |  |
| **Age at Incident ICS** | 0-4 Years | 41 (82.0%) | 131 (82.9%) | 22 (66.7%) | 2 (40.0%) | 0.023 |
|  | 5-11 Years | 7 (14.0%) | 25 (15.8%) | 10 (30.3%) | 2 (40.0%) |  |
|  | 11+ Years | 2 (4.0%) | 2 (1.3%) | 1 (3.0%) | 1 (20.0%) |  |
| **Age at Incident ICS+LABA** | 0-4 Years | 22 (44.0%) | 101 (63.9%) | 14 (42.4%) | 1 (20.0%) | 0.006 |
|  | 5-11 Years | 22 (44.0%) | 52 (32.9%) | 17 (51.5%) | 3 (60.0%) |  |
|  | 11+ Years | 6 (12.0%) | 5 (3.2%) | 2 (6.1%) | 1 (20.0%) |  |
| **PDM Risk** | Low | 10 (20.0%) | 15 (9.5%) | 7 (21.2%) | 2 (40.0%) | 0.032 |
|  | Moderate | 16 (32.0%) | 35 (22.2%) | 9 (27.3%) | 1 (20.0%) |  |
|  | High | 24 (48.0%) | 108 (68.4%) | 17 (51.5%) | 2 (40.0%) |  |
| **Allergy/Polysensitization Clusters** | 1 | 41 (82.0%) | 105 (66.5%) | 20 (60.6%) | 2 (40.0%) | 0.053 |
|  | 2 | 4 (8.0%) | 23 (14.6%) | 7 (21.2%) | 3 (60.0%) |  |
|  | 3 | 5 (10.0%) | 30 (19.0%) | 6 (18.2%) | 0 (0.0%) |  |
| **MDC Diagnosis Clusters** | 1 | 25 (50.0%) | 61 (38.6%) | 20 (60.6%) | 3 (60.0%) | 0.216 |
|  | 2 | 20 (40.0%) | 78 (49.4%) | 10 (30.3%) | 1 (20.0%) |  |
|  | 3 | 5 (10.0%) | 19 (12.0%) | 3 (9.1%) | 1 (20.0%) |  |
| **1-Year Before ICS+LABA** | SAE | 17 (34.0%) | 63 (39.9%) | 7 (21.2%) | 1 (20.0%) | 0.187 |
|  | LTRA | 22 (44.0%) | 66 (41.8%) | 12 (36.4%) | 4 (80.0%) | 0.361 |
| **Follow-up Duration (Years)** | Mean (SD) | 7.9 (4.7) | 9.4 (5.1) | 8.9 (4.7) | 10.6 (3.4) | 0.137 |

P values are based on Fisher’s exact test and Kruskal-Wallis rank sum test

**Table S3. Allergy/Allergy Sensitization by Derived HCPC Clusters**

| **Allergy/Allergy Sensitization** | **Overall (N = 249)** | **1 (N = 171)** | **2 (N = 37)** | **3 (N = 41)** | **p-value*^1^*** |
| --- | --- | --- | --- | --- | --- |
| Drugs | 127 (51.0%) | 64 (37.4%) | 30 (81.1%) | 33 (80.5%) | <0.001 |
| Eggs | 27 (10.8%) | 3 (1.8%) | 0 (0.0%) | 24 (58.5%) | <0.001 |
| Milk | 43 (17.3%) | 19 (11.1%) | 2 (5.4%) | 22 (53.7%) | <0.001 |
| Seasonal | 150 (60.2%) | 78 (45.6%) | 36 (97.3%) | 36 (87.8%) | <0.001 |
| Pets | 81 (32.5%) | 22 (12.9%) | 27 (73.0%) | 32 (78.0%) | <0.001 |
| Dust/Dust Mites | 66 (26.5%) | 6 (3.5%) | 31 (83.8%) | 29 (70.7%) | <0.001 |
| Other – General Allergens | 84 (33.7%) | 40 (23.4%) | 23 (62.2%) | 21 (51.2%) | <0.001 |
| Other-Foods | 91 (36.5%) | 29 (17.0%) | 22 (59.5%) | 40 (97.6%) | <0.001 |
| Nut | 55 (22.1%) | 16 (9.4%) | 5 (13.5%) | 34 (82.9%) | <0.001 |
| Fruit | 19 (7.6%) | 4 (2.3%) | 6 (16.2%) | 9 (22.0%) | <0.001 |
| Seafood | 24 (9.6%) | 4 (2.3%) | 2 (5.4%) | 18 (43.9%) | <0.001 |
| Insects | 34 (13.7%) | 0 (0.0%) | 25 (67.6%) | 9 (22.0%) | <0.001 |
| Smoke | 21 (8.4%) | 4 (2.3%) | 14 (37.8%) | 3 (7.3%) | <0.001 |
| Number of Allergies |  |  |  |  | <0.001 |
| Mean (SD) | 4.2 (4.2) | 1.8 (1.6) | 8.8 (3.3) | 9.8 (3.5) |  |
| Allergy Report (≥1) | 204 (81.9%) | 126 (73.7%) | 37 (100.0%) | 41 (100.0%) | <0.001 |

^1^Pearson’s Chi-squared test; Fisher’s exact test; Kruskal-Walli’s rank sum test

Seasonal allergies include allergies to fall, winter, autumn, summer, seasonal, elm, pollen, grass, hay, mold, birch, oak, pigweed, ragweed, trees and plants.

Pet allergies include allergies to cats, dogs, mice, rodents, pet dander, animal dander, animals and fur.

Dust/Dust Mites allergies include allergies to dust, dust mites, and dander.

Nut allergies include allergies to almonds, cashews, pistachios, peanuts, tree nuts, walnuts, and nuts.

Egg allergies include allergies to eggs

Seafood allergies include allergies to crab, lobster, shrimp, fish, shellfish, swordfish, tuna, scallops, and seafood.

Milk allergies include allergies to milk, and dairy.

Fruit allergies include allergies to banana, coconut, pineapple, kiwi, raspberry, strawberry, watermelon, mango and fruits.

Drug allergies include allergies to Ibuprofen, Amoxicillin, Augmentin, Tylenol, Erythromycin, Antibiotics, Penicillin, Propylene glycol and drugs.

Insect allergies include allergies to wasps, cockroaches, insect stings, bees, hornets and insects.

Smoke allergies include allergies to smoke.

Other-Food allergies include allergy to wheat, gluten, rice, pork, soy, sunflower seeds and foods.

Other-General allergens include allergy to adhesive bandages, adhesives, dyes, environment, latex, metals, narcotics, perfumes, and other.

**Table S4. MDC diagnoses (≤3 years) by HCPC Derived Clusters**

| **MDC Diagnosis Category** | **Overall (**N = 249) | **1 (**N = 110) | **2 (**N = 110) | **3 (**N = 29) | **p-value***^1^* |
| --- | --- | --- | --- | --- | --- |
| Pre-MDC | 150 (60.2%) | 24 (21.8%) | 97 (88.2%) | 29 (100.0%) | <0.001 |
| Nervous System | 73 (29.3%) | 8 (7.3%) | 40 (36.4%) | 25 (86.2%) | <0.001 |
| Eye | 62 (24.9%) | 3 (2.7%) | 37 (33.6%) | 22 (75.9%) | <0.001 |
| EMT | 137 (55.0%) | 17 (15.5%) | 93 (84.5%) | 27 (93.1%) | <0.001 |
| Respiratory | 222 (89.2%) | 85 (77.3%) | 108 (98.2%) | 29 (100.0%) | <0.001 |
| Circulatory | 83 (33.3%) | 7 (6.4%) | 50 (45.5%) | 26 (89.7%) | <0.001 |
| Digestive | 131 (52.6%) | 13 (11.8%) | 91 (82.7%) | 27 (93.1%) | <0.001 |
| Hepatic/Biliary | 12 (4.8%) | 0 (0.0%) | 0 (0.0%) | 12 (41.4%) | <0.001 |
| Musculoskeletal | 60 (24.1%) | 5 (4.5%) | 35 (31.8%) | 20 (69.0%) | <0.001 |
| Skin | 130 (52.2%) | 23 (20.9%) | 80 (72.7%) | 27 (93.1%) | <0.001 |
| Endocrine/Metabolic | 99 (39.8%) | 4 (3.6%) | 69 (62.7%) | 26 (89.7%) | <0.001 |
| Kidney/Urinary Tract | 28 (11.2%) | 5 (4.5%) | 11 (10.0%) | 12 (41.4%) | <0.001 |
| Male Reproductive | 33 (13.3%) | 4 (3.6%) | 13 (11.8%) | 16 (55.2%) | <0.001 |
| Female Reproductive | 3 (1.2%) | 1 (0.9%) | 1 (0.9%) | 1 (3.4%) | 0.481 |
| Perinatal/Postnatal | 5 (2.0%) | 0 (0.0%) | 0 (0.0%) | 5 (17.2%) | <0.001 |
| Newborn/Neonates | 123 (49.4%) | 13 (11.8%) | 81 (73.6%) | 29 (100.0%) | <0.001 |
| Blood/Immunological | 41 (16.5%) | 1 (0.9%) | 21 (19.1%) | 19 (65.5%) | <0.001 |
| Neoplasms | 6 (2.4%) | 0 (0.0%) | 4 (3.6%) | 2 (6.9%) | 0.036 |
| Infectious/Parasitic | 121 (48.6%) | 19 (17.3%) | 82 (74.5%) | 20 (69.0%) | <0.001 |
| Mental Health | 59 (23.7%) | 2 (1.8%) | 35 (31.8%) | 22 (75.9%) | <0.001 |
| Substance Use | 3 (1.2%) | 0 (0.0%) | 0 (0.0%) | 3 (10.3%) | 0.001 |
| Injuries/Poison | 43 (17.3%) | 2 (1.8%) | 33 (30.0%) | 8 (27.6%) | <0.001 |
| Burns | 5 (2.0%) | 1 (0.9%) | 3 (2.7%) | 1 (3.4%) | 0.526 |
| SDOH | 134 (53.8%) | 17 (15.5%) | 88 (80.0%) | 29 (100.0%) | <0.001 |
| Number of MDCs |  |  |  |  | <0.001 |
| Mean (SD) | 7 (5) | 2 (2) | 10 (2) | 15 (2) |  |
| MDC Diagnosis (≥1) | 230 (92.4%) | 91 (82.7%) | 110 (100.0%) | 29 (100.0%) | <0.001 |

^1^Pearson’s Chi-squared test; Fisher’s exact test; Kruskal-Wallis rank sum test
